# Global Transcriptome Analysis of the Tentacle of the Jellyfish *Cyanea capillata* Using Deep Sequencing and Expressed Sequence Tags: Insight into the Toxin- and Degenerative Disease-Related Transcripts

**DOI:** 10.1371/journal.pone.0142680

**Published:** 2015-11-09

**Authors:** Guoyan Liu, Yonghong Zhou, Dan Liu, Qianqian Wang, Zengliang Ruan, Qian He, Liming Zhang

**Affiliations:** 1 Marine Bio-pharmaceutical Institute, Second Military Medical University, Shanghai 200433, China; 2 Department of Marine Biotechnology, Faculty of Naval Medicine, Second Military Medical University, Shanghai 200433, China; 3 Department of Gynecology, Third Affiliated Hospital, Second Military Medical University, Shanghai 200433, China; Weizmann Institute of Science, ISRAEL

## Abstract

**Background:**

Jellyfish contain diverse toxins and other bioactive components. However, large-scale identification of novel toxins and bioactive components from jellyfish has been hampered by the low efficiency of traditional isolation and purification methods.

**Results:**

We performed *de novo* transcriptome sequencing of the tentacle tissue of the jellyfish *Cyanea capillata*. A total of 51,304,108 reads were obtained and assembled into 50,536 unigenes. Of these, 21,357 unigenes had homologues in public databases, but the remaining unigenes had no significant matches due to the limited sequence information available and species-specific novel sequences. Functional annotation of the unigenes also revealed general gene expression profile characteristics in the tentacle of *C*. *capillata*. A primary goal of this study was to identify putative toxin transcripts. As expected, we screened many transcripts encoding proteins similar to several well-known toxin families including phospholipases, metalloproteases, serine proteases and serine protease inhibitors. In addition, some transcripts also resembled molecules with potential toxic activities, including cnidarian CfTX-like toxins with hemolytic activity, plancitoxin-1, venom toxin-like peptide-6, histamine-releasing factor, neprilysin, dipeptidyl peptidase 4, vascular endothelial growth factor A, angiotensin-converting enzyme-like and endothelin-converting enzyme 1-like proteins. Most of these molecules have not been previously reported in jellyfish. Interestingly, we also characterized a number of transcripts with similarities to proteins relevant to several degenerative diseases, including Huntington’s, Alzheimer’s and Parkinson’s diseases. This is the first description of degenerative disease-associated genes in jellyfish.

**Conclusion:**

We obtained a well-categorized and annotated transcriptome of *C*. *capillata* tentacle that will be an important and valuable resource for further understanding of jellyfish at the molecular level and information on the underlying molecular mechanisms of jellyfish stinging. The findings of this study may also be used in comparative studies of gene expression profiling among different jellyfish species.

## Introduction

In recent decades, frequent outbreaks of jellyfish have occurred in oceans, potentially due to overfishing by humans, nutrient pollution and global warming. Jellyfish outbreaks have a strong adverse impact on marine ecological balance. However, the large amount of jellyfish biomass could be considered a valuable source of bioactive compounds. Thus, the overall development and comprehensive utilization of jellyfish have also triggered interest among many scientists.

Jellyfish bodies contain a great variety of natural bioactive components, among which the most studied are jellyfish nematocyst toxins. Nematocysts are densely located on the tentacles, and each contains a tiny dose of venom. People stung by toxic jellyfish may develop severe pain, dyspnea or even cardiorespiratory failure [1]. Many studies have explored the physicochemical properties of nematocyst toxins, which are now believed to be a type of novel protein or peptide. Jellyfish nematocyst toxins exhibit various bioactivities, such as hemolytic, enzymatic, neurotoxic, myotoxic and cardiovascular activities [2–4]. In addition to nematocyst toxins, the jellyfish body contains a wide range of novel proteins or peptides that exhibit activities such as antioxidation, antibiosis and immune reinforcing. Antioxidant activity of the giant jellyfish *Nemopilema nomurai* was observed by Kazuki [5]. We previously reported the first peroxiredoxin (Prx) and thioredoxin (Trx) genes from the jellyfish *Cyanea capillata*; both of these genes exhibit general intracellular antioxidant activity [6–7]. The jellyfish body also contains abundant collagens, which have immunostimulatory effects without inducing allergic complications [8].

Recent research has investigated and identified the bioactive components of jellyfish, particularly their toxins. However, the low efficiency of traditional isolation and purification methods has hampered the large-scale identification of novel toxins and bioactive components from jellyfish. No complete genome sequencing of jellyfish has been reported. In the absence of genome sequencing, the transcriptome represents a valuable searchable database. However, of the nearly 250 jellyfish species, only three species, *Stomolophus meleagris*, *Chironex fleckeri* and *Aurelia aurita*, have been sequenced using the transcriptome approach [9–11]. Only a small number of jellyfish sequences (54,247 ESTs and 3,795 nucleotides, as of Mar 10, 2015) have been deposited in the National Center for Biotechnology Information (NCBI) database, seriously limiting our understanding of the diverse bioactivities of this abundant marine zooplankton. Therefore, more sequence data and comprehensive analysis of jellyfish species transcriptomes are desired to explore more toxins and other bioactive components.

High-throughput next-generation sequencing technologies provide platforms to perform deep sequence analysis, which has greatly boosted comprehensive genetic research on some relatively uncharacterized species. *C*. *capillata* is one of the most common venomous jellyfish in the East China Sea. We previously demonstrated that a tentacle extract from *C*. *capillata* exhibits diverse bioactivities, including hemolytic, proteolytic, cardiovascular, cytolytic and antioxidant activities [12–14]. However, the underlying mechanisms of these bioactivities at the molecular level remain unclear. In the present study, we performed *de novo* transcriptome sequencing of the tentacle tissue of *C*. *capillata* using the Illumina HiSeq^™^ 2000 platform. A systematic bioinformatics strategy was used to conduct an in-depth and integrated analysis of this transcriptome, explore the venom composition in detail, and identify other important molecules in *C*. *capillata*.

## Material and Methods

### Jellyfish sample collection and RNA isolation

Samples of the jellyfish *C*. *capillata* were collected in July 2013 in the Sanmen Bay, East China Sea. No specific permit was required to catch *C*. *capillata*. The tentacle tissues were quickly excised manually after capture and frozen immediately in liquid nitrogen. Total RNA was isolated using TRIzol reagent (Invitrogen, CA, USA) and treated with RNase-free DNase I (Takara Biotechnology, China). RNA integrity was validated with a 2100 Bioanalyzer (Agilent Technologies, CA, USA).

### Illumina sequencing

Illumina sequencing analysis was performed according to the methods described previously [15–16]. Briefly, poly(A) mRNA was isolated using Oligo (dT) beads and interrupted to short fragments. These fragments were then transcribed into first-strand cDNA, followed by synthesis of the second strand. The synthesized cDNA products were purified using a QiaQuick PCR extraction kit (Qiagen, Valencia, CA, USA) and dissolved in EB buffer for end repair and poly(A) addition. Subsequently, the cDNA fragments were ligated to the sequencing adapters and subjected to size selection using agarose gel electrophoresis. Suitable fragments were amplified by PCR, and the cDNA library was sequenced using an Illumina HiSeq^™^ 2000 sequencer at the Beijing Genomics Institute (BGI; Shenzhen, China).

### *De novo* assembly and functional annotation

The image data output from the sequencer was transformed into sequence data called raw reads. After filtering low-quality reads and reads containing more than 5% unknown nucleotides, the sequencing adaptors were removed from the raw reads. Subsequently, the raw reads were assembled into contigs and unigenes by *de novo* assembly, which was performed with the Trinity program [17]. Finally, unigenes were aligned by BLASTx (e-value ≤ 10^−5^) to protein databases, including the NCBI non-redundant protein (Nr) database (http://www.ncbi.nlm.nih.gov), Swiss-Prot protein database (http://www.expasy.ch/sprot), Kyoto Encyclopedia of Genes and Genomes (KEGG) pathway database (http://www.genome.jp/kegg) and Cluster of Orthologous Groups (COG) database (http://www.ncbi.nlm.nih.gov/COG). Proteins with the highest sequence similarity with the given unigenes were used to determine the sequence direction, functional annotation and protein coding region. A preferential order of Nr, Swiss-Prot, KEGG and COG was followed if the results from these databases were inconsistent. If no hits were obtained for a unigene in these databases, ESTScan software [18] was used to decide the sequence direction and protein coding region. Based on Nr annotations, the Blast2GO program [19] was then used to obtain the gene ontology (GO) annotations of the unigenes, followed by GO classification using WEGO software [20]. COG and KEGG were also used to obtain functional annotations for the unigenes and analyze gene products involved in metabolism.

### Identification of toxin-like transcripts

According to our previous studies of *C*. *capillata* and other reports on various jellyfish, the toxic effects of jellyfish venom primarily include vasoconstriction, hemorrhage, and hemolytic and cardiovascular toxicities. To explore the underlying molecular mechanisms of these toxic actions and identify as many putative toxin transcripts in *C*. *capillata* as possible, three strategies were used. First, we compared the unigene sequences to a toxin database in Swiss-Prot, Tox-Prot (http://www.uniprot.org/program/Toxins), based on sequence homology. Second, to make the screening more complete, we also manually searched the annotations of the unigenes under the term ‘toxin’ or ‘venom’. Third, according to the symptoms after jellyfish envenomation, we referred to many previous reports on venomous components in different types of venomous animals, such as snakes, scorpions, spiders, wasps and sea anemones, to construct a reference guide of estimated toxin-like transcripts.

### Analysis of transcripts related to degenerative diseases

Sequences encoding proteins associated with degenerative diseases, including Huntington’s disease (HD), Alzheimer's disease (AD) and Parkinson's disease (PD), were identified by BLAST results against the Nr database, with a cut-off value of e-value ≤ 10^−5^.

### Bioinformatics analyses and alignments

Bioinformatics analyses were performed following methods we have described previously [7]. Briefly, the ORF Finder program (http://www.ncbi.nlm.nih.gov/gorf/gorf.html) and SignalP 4.1 Server (http://www.cbs.dtu.dk/services/SignalP/) were used to search for the open reading frames and signal peptides, respectively, in the sequences. Sequence alignments were performed using the ClustalW2 program (http://www.ebi.ac.uk/Tools/msa/clustalw2/). Phylogenetic analysis was performed using MEGA 4 software.

## Results and Discussion

### Illumina sequencing and reads assembly

A total of 54,109,750 raw reads were obtained using the Illumina HiSeq^™^ 2000 platform. After cleaning and removing dirty reads containing adapters, unknown or low quality bases, a total of 51,304,108 clean reads corresponding to more than 4.61 billion clean nucleotides were generated (Table 1). The average length of the clean reads was 90 bp, consistent with the sequencing capacity of the Illumina device. The Q20 percentage, N percentage and GC percentage were 99.15%, 0.02% and 40.26%, respectively. The original sequencing data for the clean reads have been deposited in the NCBI Sequence Read Archive (SRA) database (accession number SRP056566).

**Table 1:** Summary of the *C*. *capillata* tentacle transcriptome.

**Sequencing statistics**	
Number of raw reads	54,109,750
Number of clean reads	51,304,108
Total clean nucleotides (bp)	4,617,369,720
Average length of clean reads (bp)	90
Q20 (%)	99.15
N (%)	0.02
GC (%)	40.26
**Assembly statistics**	
**Contigs**	
Total number	125,058
Total length (bp)	32,284,466
Mean length (bp)	258
N50 length	313
**Unigenes**	
Total number	50,536
Total length (bp)	25,414,791
Mean length (bp)	503
N50 length	644
Total consensus sequences	50,536
Distinct clusters	861
Distinct singletons	49,675

Using the Trinity program, a total of 125,058 contigs corresponding to more than 32 million nucleotides were assembled from the short reads. Among these assembled contigs, 90.50% (113,176) were between 100 and 500 bp in length, 5.96% (7,451) were between 500 and 1000 bp, 2.99% (3,740) were between 1000 and 2000 bp, and 0.55% (691) were more than 2000 bp. Finally, the contigs were connected, and 50,536 unigenes were generated, with a mean length of 503 bp. Although most unigenes (36,224, 71.68%) were between 100 and 500 bp, we obtained 14,312 unigenes that were greater than 500 bp in length. The length distributions of these assembled contigs and unigenes are shown in S1 Fig. Protein coding sequences (CDS) of all assembled unigenes were predicted. A total of 20,892 potential CDSs were identified by BLAST searches, and 5,817 CDSs were predicted by ESTScan.

Transcriptome analysis using Illumina sequencing technology is one of the most popular tools for gene discovery, and it has recently been applied to several species that lack genomic sequence information [21–23]. Therefore, the transcriptome data for *C*. *capillata* obtained here will enrich the sequence information previously available for jellyfish in public databases. In addition, this transcriptome could provide more detailed and general genetic data to facilitate large-scale discovery and rapid characterization of novel important genes from jellyfish.

### Functional annotation and classification

**Fig 1:**
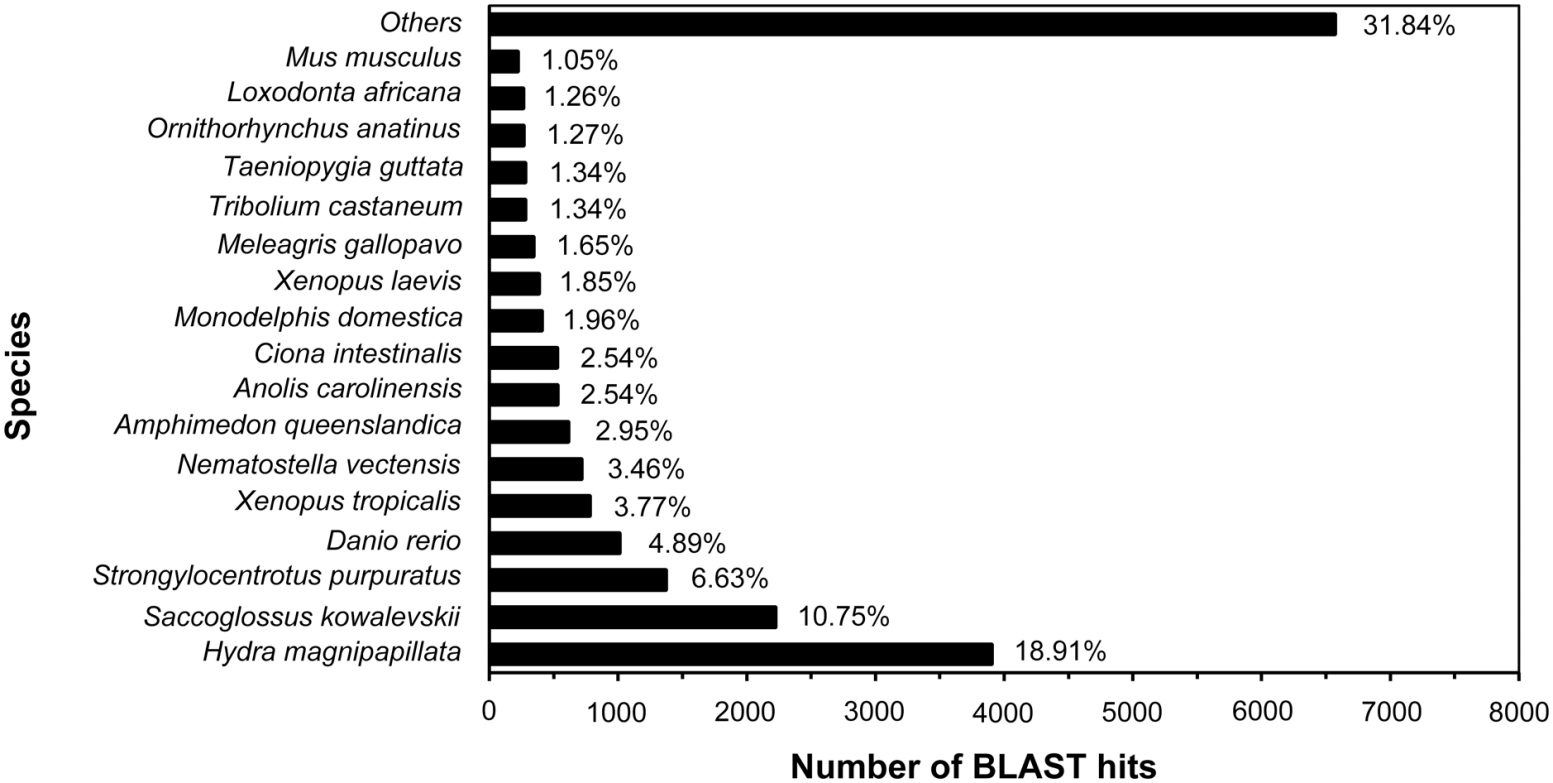
Top-hit species distribution of the BLASTx matches of the unigenes. The species distributions of the top BLASTx hits against the Nr database were analyzed (cut-off e-value < 10^−5^). Species with proportions of greater than 1% are shown.

To obtain functional annotations of the predicted proteins, the assembled unigenes were used as a query for Blastx alignments to several public protein databases, including the Nr, Swiss-Prot, KEGG and COG databases. An e-value < 10^−5^ was used as a cut-off for confident homologue detection. Of a total of 50,536 unigenes, hits were obtained for 20,629 (40.8%) in the Nr database. This large percentage of hits was anticipated. In addition, hits were obtained for 17,006 (33.7%), 13,663 (27.0%) and 6,202 (12.3%) unigenes in the Swiss-Prot, KEGG and COG protein databases, respectively. We also aligned all of the unigenes by Blastn to the Nt nucleotide database, and 4,878 (9.7%) had significant hits. The functional annotations by BLAST searches of the 50,536 unigenes are presented in S1 Table. The e-value distributions were also calculated. Among the 20,629 unigenes that had homologous proteins in the Nr protein database, more than half of the matched sequences (11,793, 57.2%) had an e-value ranging between 1e^-10^ and 1e^-50^, and 6.6% (1,368) had homology with an e-value smaller than 1e^-100^, indicating strong reliability of the alignment. To sum up, a total of 21,357 (42.3%) unigenes were significantly similar to the unique protein accessions in each of the above databases. However, more than half of the unigenes had no significant matches due to the limited sequence information for jellyfish and their closely related species or partly due to a high number of species-specific transcripts and novel sequences. The species distributions of the top BLAST hits against the Nr database were also analyzed. The assembled unigenes had the greatest number (3,900, 18.91%) of matches with *Hydra magnipapillata*. Because jellyfish and hydra are both included in the phylum of Cnidaria, sequence similarities are likely to be highest with closely related species. The next species were *Saccoglossus kowalevskii* (2,218, 10.75%), *Strongylocentrotus purpuratus* (1.368, 6.63%), *Danio rerio* (1,008, 4.89%), *Xenopus tropicalis* (778, 3.77%) and another typical species of Cnidaria, *Nematostella vectensis* (713, 3.46%). Other species with proportions of greater than 1% are also shown in Fig 1. However, few matches corresponded to jellyfish species, which might also be due to the limited number of jellyfish protein sequences available in the database.

GO annotation based on sequence homology was used to determine the GO terms of the unigenes. A total of 6,657 (13.2%) unigenes were categorized into at least one group of 49 sub-categories of three independent ontology categories (S2 Fig). Among the 26 sub-categories of “biological process”, “cellular process” (3412 unigenes) was the most dominant group, followed by “metabolic process” (2578 unigenes) and “biological regulation” (1467 unigenes), indicating that many extensive metabolic activities and rapid growth may occur in the tentacle of *C*. *capillata*. For the “cellular component” category, the most representative of the assignments were “cell” (4373 unigenes), “cell part” (4041 unigenes) and “organelle” (2701 unigenes). Within the 12 groups corresponding to “molecular function”, the dominant distributions were from “binding” (2895 unigenes) and “catalytic activity” (2718 unigenes). These GO annotations represented the general gene expression profile characteristics for the tentacle of *C*. *capillata*. To further predict and classify possible functions of the unigenes, COG assignments were used (S3 Fig). The category of “general function prediction only”, which contained 2,079 unigenes (33.52%), was the largest group, followed by “replication, recombination and repair” (898, 14.48%), “translation, ribosomal structure and biogenesis” (841, 3.94%) and “posttranslational modification, protein turnover, chaperones” (769, 12.40%). The categories of “extracellular structures” (3, 0.05%) and “nuclear structure” (8, 0.13%) were the smallest groups. The GO and COG functional classifications thus provided valuable and detailed information for investigating specific processes and functions in jellyfish tentacle.

**Fig 2:**
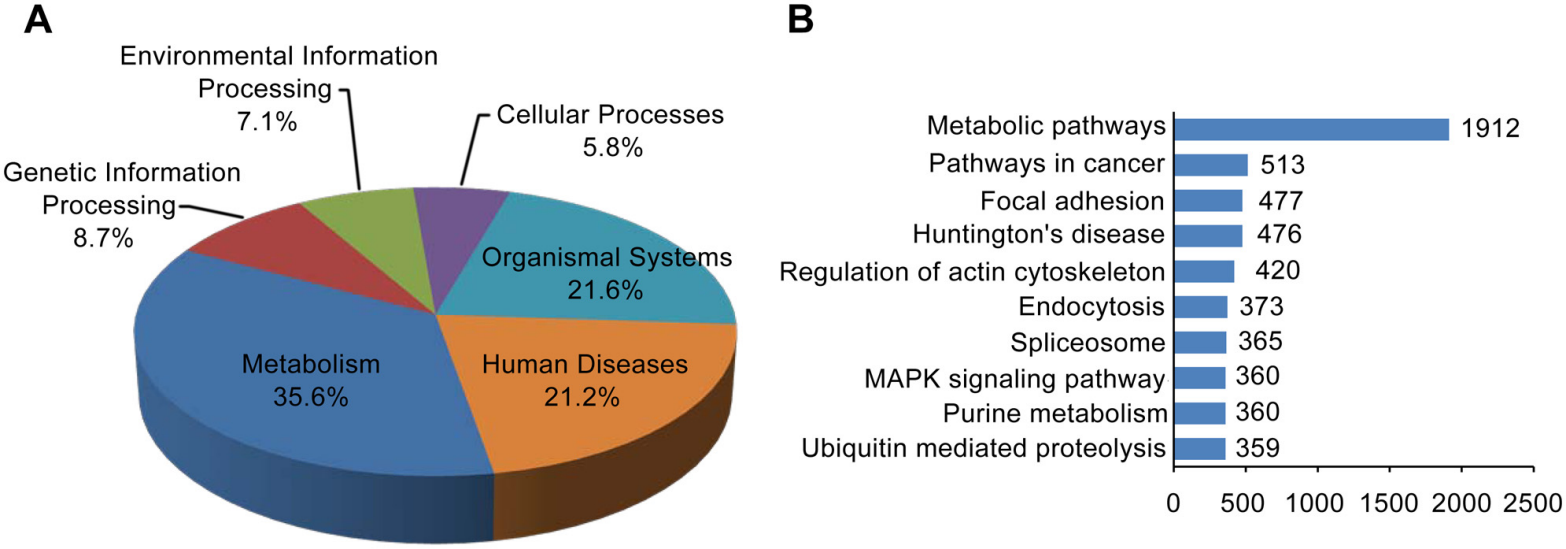
Categorization of unigenes into KEGG biochemical pathways. A total of 13,663 unigenes were assigned to 241 KEGG pathways belonging to six categories. (A) The percentage of the pathway amount in each category is shown. (B) The ten largest groups with KEGG database annotation. The x-axis indicates the number of annotated unigenes.

To identify the active metabolic pathways in the tentacle of *C*. *capillata*, the annotated unigenes were mapped to KEGG pathways. A total of 13,663 unigenes were assigned to 241 KEGG pathways consisting of the categories of “metabolism” (86 pathways), “genetic information processing” (21 pathways), “environmental information processing” (17 pathways), “cellular processes” (14 pathways), “organismal systems” (52 pathways) and “human diseases” (51 pathways) (Fig 2A). Among the mapped pathways, “metabolic pathways” contained 1,912 unigenes (13.99%) and was obviously larger than the other groups, such as “pathways in cancer” (513, 3.75%), “focal adhesion” (477, 3.49%), “‘Huntington’s disease” (476, 3.48%) and “regulation of actin cytoskeleton” (420, 3.07%). The top 10 pathways are shown in Fig 2B, and all pathways are summarized in S2 Table.

In summary, based on *de novo* sequencing and in-depth analysis, we obtained a well-annotated transcriptome that could provide valuable information for identifying novel genes and investigating specific metabolic pathways in the tentacle of the jellyfish *C*. *capillata*.

### Analysis of putative toxin transcripts in *C*. *capillata*

Interest in the biological activities of jellyfish toxins has increased considerably in recent years. However, due to the technical difficulties of obtaining toxins and their labile nature, the molecular mechanisms of jellyfish toxins remain largely unknown. Few molecules in jellyfish venom have been described, hampering the exploration of the pathophysiology of jellyfish envenomation and proper patient care. Many studies have demonstrated that jellyfish venom consists of a complex mixture of proteins and peptides with toxic or enzymatic actions [13,24–26]. Therefore, one of the main goals of the present study is to identify as many putative toxin transcripts in *C*. *capillata* as possible and build a reference database of toxin sequences to facilitate the future analysis of other jellyfish species.

#### 1. Venom constituents similar to known toxin families

Based on the symptoms of jellyfish stings, we identified a large number of transcripts encoding proteins similar to several well-known toxin families that have been widely described in various venomous animals. These toxin transcripts are further classified and summarized in Table 2.

**Table 2:** Annotation of transcripts similar to known toxin families.

**Sequence ID**	ORF^a^/Length(bp)	Signal peptide^b^	Annotation	Best matched species/Maximum identify (%)	E-value
**Phospholipases**					
**Unigene7860**	F/784	Y	Group 3 secretory phospholipase A2-like	*Acyrthosiphon pisum*/46	3E-19
**Unigene9375**	N/289	U	Group 3 secretory phospholipase A2-like	*Gallus gallus*/45	2E-09
**Unigene17685**	F/1945	Y	Group XV phospholipase A2-like	*Aplysia californica*/47	6E-104
**Unigene19288**	F/626	Y	Phospholipase A2	*Rhopilema esculentum*/43	2E-39
**Unigene27084**	F/662	Y	Phospholipase A2	*Rhopilema esculentum*/57	1E-52
**Unigene35028**	F/804	Y	Phospholipase A2	*Rhopilema esculentum*/36	6E-19
**Unigene37728**	F/562	N	Phospholipase A2	*Rhopilema esculentum*/49	2E-15
**Unigene28609**	N/284	U	85/88 kDa calcium-independent phospholipase A2	*Hydra vulgaris*/59	1E-11
**Unigene47062**	N/546	U	85/88 kDa calcium-independent phospholipase A2	*Hydra vulgaris*/54	3E-55
**Unigene41691**	N/967	U	Calcium-independent phospholipase A2-gamma-like	*Lepisosteus oculatus*/52	3E-94
**Unigene4792**	N/305	U	Phospholipase D1-like	*Hydra vulgaris*/62	5E-29
**Unigene23083**	N/277	U	Phospholipase D1	*Ixodes scapularis*/46	3E-17
**Unigene48846**	N/324	U	Phospholipase D1-like	*Metaseiulus occidentalis*/45	3E-12
**Unigene30910**	N/435	U	Phospholipase D3-like	*Aplysia californica*/54	9E-36
**Unigene48788**	N/255	U	Phospholipase D3	*Ascaris suum*/56	9E-23
**Metalloproteinases**					
**Unigene18144**	F/1437	N	Matrix metalloproteinase-14	*Camelus ferus*/33	2E-24
**Unigene34794**	N/670	N	Matrix metalloproteinase-14	*Ascaris suum*/43	1E-31
**Unigene39586**	N/1639	N	Matrix metalloproteinase-1	*Drosophila melanogaster*/43	2E-16
**Unigene9370**	N/215	U	Matrix metalloproteinase-14-like	*Saccoglossus kowalevskii*/70	1E-13
**Unigene18671**	F/2463	Y	Matrix metalloproteinase	*Hydra vulgaris*/38	8E-98
**Unigene19077**	F/2544	Y	Matrix metalloproteinase	*Hydra vulgaris*/39	1E-98
**Unigene18967**	N/1127	Y	Matrix metalloproteinase-24-like	*Hydra vulgaris*/44	9E-57
**Unigene21910**	N/836	Y	Matrix metalloproteinase-9-like	*Strongylocentrotus purpuratus*/37	1E-12
**Unigene41786**	F/1953	Y	Matrix metalloproteinase-25-like	*Hydra vulgaris*/36	6E-93
**Unigene6794**	F/2042	N	Matrix metalloproteinase	*Hydra vulgaris*/42	1E-116
**Unigene13316**	N/413	U	Matrix metalloproteinase-28	*Sarcophilus harrisii*/48	3E-11
**Unigene8341**	F/1412	Y	Zinc metalloproteinase nas-4	*Hydra vulgaris*/53	6E-76
**Unigene19338**	F/1253	Y	Zinc metalloproteinase nas-4	*Hydra vulgaris*/39	3E-58
**Unigene17414**	F/1901	Y	Zinc metalloproteinase nas-13-like	*Hydra vulgaris*/50	1E-59
**Unigene18943**	F/2096	Y	Zinc metalloproteinase nas-13-like	*Hydra vulgaris*/40	7E-54
**Unigene7643**	F/1098	Y	Zinc metalloproteinase nas-15-like	*Hydra vulgaris*/43	6E-66
**Unigene18427**	F/1440	Y	Astacin 3	*Hydractinia echinata*/49	6E-86
**Serine proteases**					
**Unigene17637**	N/1100	U	Serine protease 1	*Aurelia aurita*/52	4E-80
**Unigene8850**	F/1033	Y	Serine protease 1	*Aurelia aurita*/68	7E-134
**Unigene34706**	N/834	U	Serine protease 1	*Aurelia aurita*/70	7E-120
**Unigene10683**	N/459	U	Serine protease 1	*Aurelia aurita*/65	1E-66
**Unigene10980**	N/340	U	Serine protease 2	*Aurelia aurita*/59	1E-41
**Unigene23736**	N/324	U	Serine protease 2	*Aurelia aurita*/50	1E-32
**Unigene18480**	N/2005	U	Transmembrane protease serine 6-like	*Hydra vulgaris*/37	8E-55
**Unigene19320**	F/2208	Y	Chymotrypsin-like elastase family member 2A-like	*Hydra vulgaris*/35	3E-49
**Unigene41758**	F/1540	Y	Chymotrypsin-like elastase family member 2A-like	*Hydra vulgaris*/39	6E-64
**Unigene40159**	N/262	U	Chymotrypsin-like elastase family member 1-like	*Hydra vulgaris*/50	1E-23
**Unigene1406**	N/756	U	Chymotrypsin-like elastase family member 3B-like	*Hydra vulgaris*/50	1E-70
**Unigene44510**	F/609	N	Chymotrypsin-like elastase family member 3B-like	*Hydra vulgaris*/49	2E-48
**Unigene10156**	N/232	U	Chymotrypsin-C-like	*Meleagris gallopavo*/45	5E-08
**Unigene15770**	F/789	N	Chymotrypsin-like protease CTRL-1-like	*Danio rerio*/37	1E-35
**Unigene42233**	F/1362	N	Serine proteinase stubble-like	*Bombus impatiens*/27	2E-17
**Serine proteinase inhibitor**					
**Unigene17288**	F/1137	N	Kazal-type serine proteinase inhibitor 4	*Procambarus clarkii*/51	1E-05
**Unigene23109**	F/1006	N	Kazal-type serine proteinase inhibitor 1	*Fenneropenaeus chinensis*/34	2E-31
**Unigene35150**	F/1748	N	Kazal-type serine proteinase inhibitor 1	*Fenneropenaeus chinensis*/35	4E-42
**Unigene38364**	F/1109	Y	Kazal-type serine proteinase inhibitor	*Pacifastacus leniusculus*/48	2E-18
**Unigene40624**	F/420	N	Kunitz-type serine protease inhibitor 3	*Chlorocebus sabaeus*/59	4E-15
**Unigene18473**	F/546	Y	Kunitz domain protein	*Ixodes scapularis*/53	9E-14
**Unigene23096**	F/764	N	Papilin-like	*Xiphophorus maculatus*/34	4E-11
**Unigene40962**	N/1476	U	Serine peptidase inhibitor, Kunitz-type 1 b	*Danio rerio*/36	8E-09
**Unigene42995**	F/1779	Y	Serpin B4	*Lctidomy tridecemlineatus*/33	1E-61
**Unigene17438**	F/2066	N	Serine protease inhibitor	*Cyanea capillata*/66	2E-140
**Unigene18138**	F/1576	Y	Leukocyte elastase inhibitor-like	*Condylura cristata*/39	4E-70
**Unigene18959**	F/1653	Y	Proteinase inhibitor 14 serpin	*Pedosphaera parvula*/36	6E-69
**Unigene41584**	F/1356	Y	Serpin B4	*Lctidomys tridecemlineatus*/40	4E-71

^a^ Full-length and not full-length open reading frames (ORFs) are indicated by “F” and “N”, respectively.

^b^ Transcripts with or without a signal peptide are indicated by “Y” and “N”, respectively, whereas transcripts in which the presence of a signal peptide was unclear are indicated by “U” (Unknown).

Phospholipases: We identified many members and isoforms of phospholipase A2s (PLA2s) and phospholipase D (PLD) in the *C*. *capillata* tentacle transcriptome. PLA2s are the most common type of phospholipases identified in the venom of various toxic animals, such as snakes, scorpions and ants [27–30]. High levels of PLA2 activity have also been described in the tentacles of scyphozoan and cubozoan species [31]. PLA2s were also recently reported to be one of the most abundant toxins in the venom of the jellyfish *S*. *meleagris* [9]. In this study, we identified 10 unique transcripts encoding PLA2s in the tentacle of *C*. *capillata* (Table 2), and six of the 10 transcripts had clear open reading frames. Generally, hemolysis is considered to be the direct result of PLA2s and hemolysin, which can interact with ion channels, membrane proteins and membrane ion pumps. We also previously observed both *in vitro* and *in vivo* hemolysis of the tentacle extract from *C*. *capillata* [12]. Hemolytic activity is considered one of the most common biological activities of jellyfish venom [32–33]. In addition to hemolytic effects, venom PLA2s can also mediate several other toxic responses, such as cytotoxicity, cardiotoxicity, neurotoxicity, myotoxicity, edema and blood coagulation disturbance [34–35].

In addition to PLA2s, we also identified five transcripts for PLDs in the transcriptome. This is the first report of PLDs in jellyfish species. PLD has been reported to act as a dermonecrotic factor in the venom of brown spiders and plays a role in the necrotic effect and severe inflammatory response [36]. Interestingly, jellyfish venom exhibits an obvious dermonecrotic effect [37–38]. *C*. *capillata* venom can induce dermonecrotic lesions in the skins of rats and Guinea pigs [39]. Thus, the presence of PLDs in the *C*. *capillata* tentacle transcriptome is a significant finding that may help to advance the discovery of the dermonecrotic mechanism of jellyfish venom.

Metalloproteases: Two types of metalloproteases were identified: matrix metalloproteinases and astacin-like metalloproteases (Table 2). The identification of these diverse metalloprotease transcripts is not surprising, because metalloproteases have been described as the central toxic component in various venomous animals [40–42]. In snake venoms, metalloprotease toxins are predominantly responsible for local pathological effects, such as tissue damage, necrosis and hemorrhage [43]. In this study, 11 unique transcripts encoding matrix metalloproteinases families, including matrix metalloproteinase-14, -1, -9, -24 and -25, were identified, and three of the 11 transcripts aligned best with metalloproteinase-14. This identification supports a previous report describing metalloprotease-14 as the main venom-derived proteins in the jellyfish *Nemopilema nomurai* [44].

**Fig 3:**
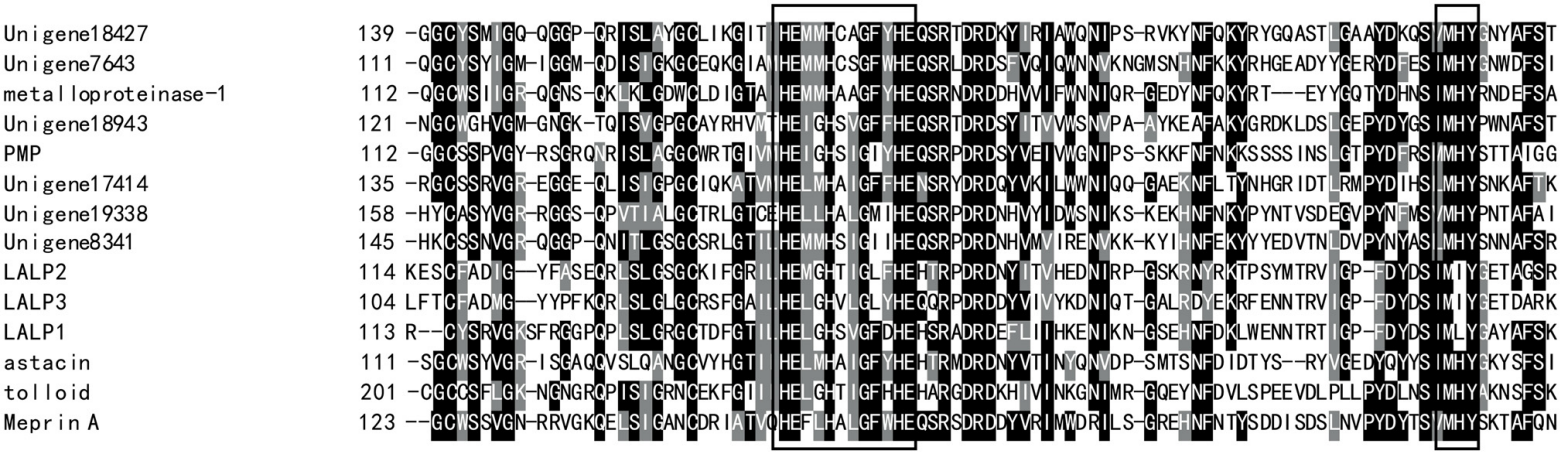
Multiple sequence alignment of astacin-family enzymes. The deduced amino acid sequences of unigenes 18427, 7643, 18943, 17414, 19338 and 8341 were aligned with *Loxosceles intermedia* LALP 1, 2 and 3 (A0FKN6, C9D7R2 and C9D7R3, respectively), metalloproteinase 1 from *H*. *vulgaris* (AAA92361), astacin from *A*. *astacus* (CAA64981), PMP1 from *Podocoryna carnea* (CAA06314), tolloid from *Drosophila melanogaster*(AAF56329) and Meprin A from *Homo sapiens* (AAI36560). Sequence accession numbers are shown in brackets. The characteristic astacin family signatures are boxed. Black and grey indicate amino acids that are identical or highly conserved, respectively, across all aligned sequences. Absent amino acids are indicated by dashes (-) to improve the alignment.

In addition, six transcripts for the astacin-like metalloproteases were also identified. Astacin-like metalloproteases have been reported to be the main components of *Loxosceles* toxins [45]. Multiple alignment analysis revealed that all of these deduced amino acid sequences contain the astacin family signatures HEXXHXXGXXHE (enzymatic catalytic domain) and MXY (Met-turn), similar to the astacin-like toxins in the *Loxosceles* genus and other astacin family-related members (Fig 3). *Loxosceles* astacin toxins can degrade extracellular matrix proteins and assist the spread of other venom components. However, the study of astacin-like metalloproteases in jellyfish venom remains in its infancy. Scyphozoan jellyfish venom has significant gelatinolytic, caseinolytic, and fibrinolytic activities [46]. Astacin-like metalloproteases were also recently identified as an important component of the venoms of *S*. *meleagris* and *N*. *nomurai* jellyfish [9,44]. Based on these findings, these astacin-like metalloproteases identified from *C*. *capillata* likely also play an important role in the pathological processes of *C*. *capillata* envenomation.

Serine proteases: Serine proteases are the best-characterized venom component. In this study, we identified 15 unique sequences of this family (Table 2), but only six were confirmed to have a complete ORF due to their high molecular weights. Among the 15 transcripts, six sequences were significantly homologous to serine proteases from the jellyfish *Aurelia aurita*. Serine proteases have been described in the venoms of snakes, spiders and scorpions [28,36,47]. Generally, this toxin family affects a wide array of physiological functions, including platelet aggregation and fibrinolytic pathways [48]. They can also play a role in post-translation modification and spreading other toxins [49]. However, the exact role of serine proteases in jellyfish envenomation remains to be clarified.

**Fig 4:**
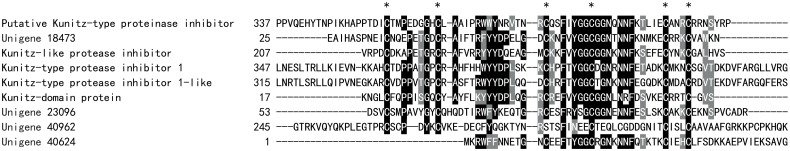
Alignment of the conserved sequence motifs of Kunitz-type inhibitors. The deduced amino acid sequences of unigenes 18473, 23096, 40962 and 40624 were aligned with known Kunitz-type inhibitors, including *Ixodes scapularis* Kunitz-domain protein (XP_002435213), *Crassostrea gigas* putative Kunitz-type proteinase inhibitor (EKC39386), *Latrodectus hesperus* Kunitz-like protease inhibitor (ADV40132), *Astyanax mexicanus* Kunitz-type protease inhibitor 1-like (XP_007252976) and *Danio rerio* Kunitz-type protease inhibitor 1 (AAI63937). The six highly conserved cysteine residues are indicated by asterisks.

Serine protease inhibitors: Several serine protease inhibitors were identified in this study, including Kazal-type (4 transcripts) and Kunitz-type (KUNs) (4 transcripts). The Serpin family (5 transcripts) was also identified (Table 2). Serine protease inhibitors have been widely found in the venoms of many well-known toxic animals [50–52]. However, few toxins of this type have been reported in jellyfish venom. Among this toxin family, Kunitz-type inhibitors have been commonly observed in snake venoms and inhibit both serine proteases and calcium ion channels. They are characterized by three disulfide bonds belonging to a highly conserved motif of C-8X-C-15X-C-4X-YGGC-12X-C-3X-C [50]. In this study, multiple alignment analysis demonstrated that compared with other typical Kunitz-type inhibitors, most of these identified Kunitz-type inhibitor transcripts possess a native Kunitz architecture (Fig 4). The function of serine protease inhibitors in the various venoms has been suggested to be primarily related to the protection of toxin integrity. Additionally, this toxin family may play a role in various physiological processes, such as blood coagulation, fibrinolysis and host defense. However, whether serine protease inhibitors in jellyfish venoms have a similar function remains to be explored, and further investigations are needed.

#### 2. Other possible venom components

In addition to the well-known types of venom toxin families described above, some transcripts resembled putative molecules with potential toxic activities reported in other venomous animals. Moreover, most of these sequences have not yet been described in jellyfish species. Thus, these transcripts were classified as ‘other possible venom components’, and the main features of these molecules are presented in Table 3.

**Table 3:** Additional putative toxin transcripts identified by similarity searches.

Sequence ID	ORF^a^/Length(bp)	Signal peptide^b^	Identification (best matched species)	E-value	Possible venom function
Unigene6213	N/307	U	Toxin TX2(*Aurelia aurita*)	2E-06	Hemolytic activity and pore-forming action
Unigene8172	F/2294	N	Venom dipeptidyl peptidase 4 isoform X2 (*Nasonia vitripennis*)	2E-109	Endopeptidases
Unigene41308	F/961	N	Venom toxin-like peptide-6 (*Mesobuthus eupeus)*	4E-07	Unknown
Unigene41678	N/566	N	Plancitoxin-1-like (*Hydra vulgaris*)	5E-59	Potently hepatotoxic
Unigene33521	N/523	Y	Plancitoxin-1-like (*Hydra vulgaris*)	9E-17	Potently hepatotoxic
Unigene4165	N/327	U	Venom protein Ci-120 (*Chelonus inanitus*)	6E-30	Proteoglycan metabolism
Unigene49242	N/330	N	Venom protein Ci-80a (*Chelonus inanitus*)	4E-42	Peptidase
Unigene17029	F/734	N	Translationally controlled tumor protein homology, TCTP (*Ixodes scapularis*)	3E-23	Histamine releasing factor
Unigene7297	F/1784	N	Angiotensin-converting enzyme-like (*Hydra vulgaris*)	1E-178	Releasing vasoconstrictive peptides
Unigene29942	N/231	U	Endothelin-converting enzyme 1-like (*Hydra vulgaris*)	5E-06	Releasing vasoconstrictive peptides
Unigene25995	N/313	U	Neprilysin-like (*Myotis davidii*)	5E-30	Endopeptidases and potent neurotoxicity
Unigene1506	N/672	U	Ectonucleotide pyrophosphatase/phosphodiesterase family member 2-like (*Hydra vulgaris*)	6E-22	Hemostatic disturbance
Unigene21452	N/1661	N	Ectonucleotide pyrophosphatase/phosphodiesterase family member 5 (*Zonotrichia albicollis*)	3E-101	Hemostatic disturbance
Unigene28395	N/221	U	Ectonucleotide pyrophosphatase/phosphodiesterase family member 1 (*Columba livia*)	4E-05	Hemostatic disturbance
Unigene39268	N/1821	U	Ectonucleotide pyrophosphatase/phosphodiesterase family member 4-like (*Hydra vulgaris*)	6E-73	Hemostatic disturbance
Unigene17070	F/1758	Y	Vascular endothelial growth factor A-like (*Acyrthosiphon pisum*)	7E-09	Inducing vasodilation and increasing vascular permeability
Unigene17673	F/1989	Y	Vascular endothelial growth factor A-like (*Hydra vulgaris*)	2E-17	Inducing vasodilation and increasing vascular permeability
Unigene39767	F/1870	N	Vascular endothelial growth factor toxin (*Lepeophtheirus salmonis*)	2E-06	Unknown
Unigene17310	F/1239	Y	Vascular endothelial growth factor D (*Crassostrea gigas*)	3E-05	Unknown
Unigene40990	N/498	U	Lysosomal acid lipase/cholesteryl ester hydrolase-like (*Hydra vulgaris*)	2E-48	Unknown
Unigene17402	F/2567	Y	Lysosomal acid lipase/cholesteryl ester hydrolase-like (*Hydra vulgaris*)	1E-160	Unknown
Unigene17303	F/1764	Y	Lysosomal acid lipase/cholesteryl ester hydrolase-like (*Hydra vulgaris*)	5E-147	Unknown
Unigene9684	N/406	U	Alkaline phosphatase D domain containing protein (*Acanthamoeba castllanii*)	2E-36	Unknown
Unigene41552	F/1246	N	Dipeptidyl peptidase 3-like (*Saccoglossus kowalevskii*)	3E-97	Inducing hypotension
Unigene8247	F/1977	N	Ectonucleoside triphosphate diphosphohydrolase 1 (*Falco peregrinus*)	7E-65	Potential inhibitor of platelet aggregation
Unigene19363	F/1872	N	Ectonucleoside triphosphate diphosphohydrolase6 (*Danio rerio*)	5E-94	Potential inhibitor of platelet aggregation

^a^ Full-length and not full-length open reading frames (ORFs) are indicated by “F” and “N”, respectively.

^b^ Transcripts with or without a signal peptide are indicated by “Y” and “N”, respectively; transcripts in which the presence of a signal peptide was unclear are indicated by “U” (Unknown).

**Fig 5:**
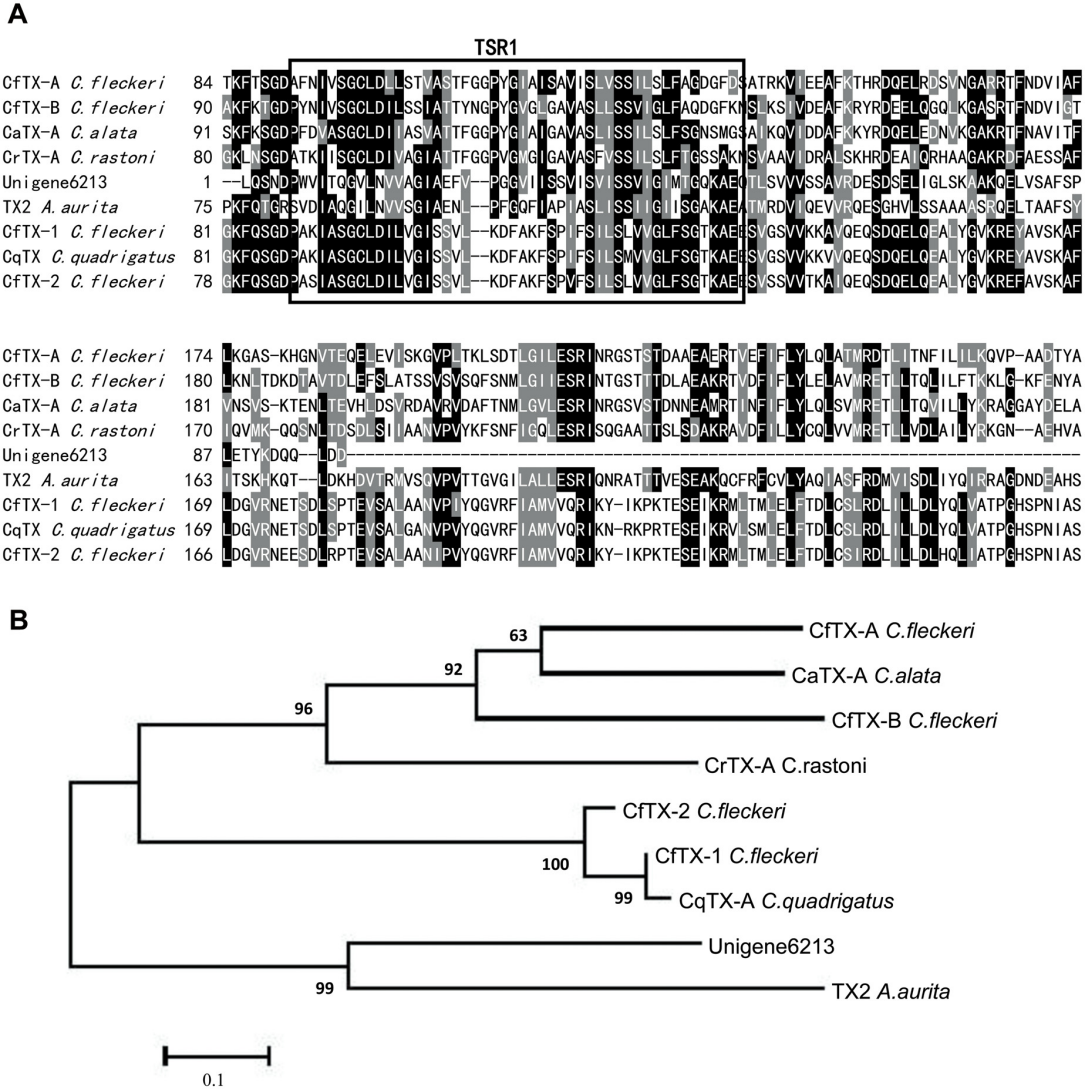
**(A) Multiple sequence alignment of the amino acid sequence of unigene 6213 with other known CfTX-like cnidarian toxins.** The abbreviations and sequence accession numbers for the aligned sequences are as follows: *Aurelia aurita* TX2 (AFK76349), *Chiropsalmus quadrigatus* CqTX-A (BAB82520), *Carybdea rastonii* CrTX-A (BAB12728), *Carybdea alata* CaTX-A (BAB12727), and *Chironex fleckeri* CfTX-A, CfTX-B, CfTX-1 and CfTX-2 (AFQ00676, AFQ00677, ABS30940 and ABS30941, respectively). The putative highly conserved transmembrane spanning region (TSR1) in the N-terminal sequences of this toxin family is boxed. (B) Phylogenetic relationships of the CfTX-like cnidarian toxins.

In this study, we identified a transcript (Unigene6213) with significant similarity to the N-terminal sequences of a family of known jellyfish toxins, including TX2 isolated from *Aurelia aurita*, CfTX-1,2 and CfTX-A,B from *Chironex fleckeri*, CqTX-A from *C*. *quadrigatus*, CrTX-A from *C*. *rastoni* and CaTX-A from *C*. *alata* [53–54]. The multiple sequence alignment and phylogenetic analysis of these similar jellyfish toxins are presented in Fig 5. This toxin family is primarily associated with potent hemolytic activity and pore-forming action [53,55]. As shown in Fig 5, a putative transmembrane spanning region (TSR1) that is highly conserved in the N-terminal sequences of this toxin family is also present in the predicted amino acid range (6–50) of Unigene6213. The presence of the transmembrane spanning region in these jellyfish toxins is very important because it may play a role in the pore-forming process [50]. Therefore, Unigene6213 is likely a new member of this family of jellyfish toxins, even though it does not contain a full-length ORF. Further research is needed to determine the full-length sequence, structure and biological role of this putative toxin in *C*. *capillata*.

**Fig 6:**
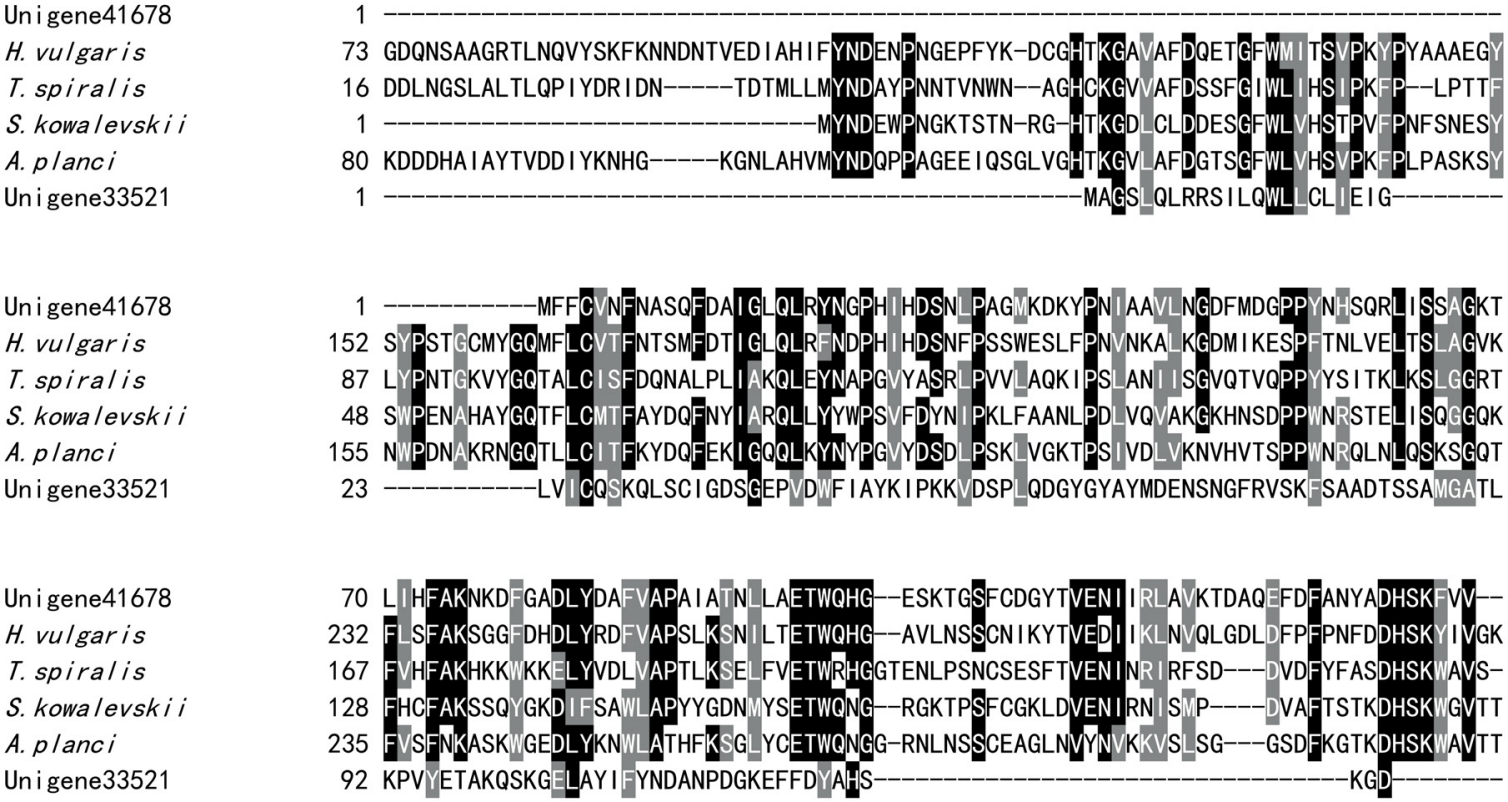
Alignment of plancitoxin-1-like proteins. The aligned sequences are as follows: *Acanthaster planci* plancitoxin 1 (BAD12432), *Saccoglossus kowalevskii* plancitoxin 1-like (XP_006823536), *T*. *spiralis* plancitoxin1-like protein (AHM10158) and *Hydra vulgaris* plancitoxin 1-like (XP_004209299). Black and gray indicate amino acids that are identical or highly conserved, respectively, across all aligned sequences.

**Fig 7:**
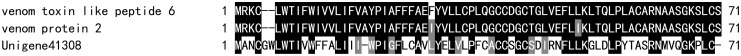
Alignment of the amino acid sequence of unigene 41308 with *Mesobuthus eupeus* venom toxin-like peptide-6 and venom protein 2. The GenBank accession numbers for venom toxin-like peptide-6 and venom protein 2 are ABR21046 and ABR21036, representatively. Black and gray indicate amino acids that are identical or highly conserved across all aligned sequences, respectively.

In addition to the homologues of known jellyfish toxins, we identified a number of toxin-like transcripts similar to some unique venom components isolated from other venomous animals. Two putative toxin transcripts with high similarity to plancitoxin-1-like, a gene closely related to plancitoxin-1 from the crown-of-thorns starfish *Acanthaster planci*, were also identified (Fig 6). Plancitoxin-1 is one of the few known toxic DNase II proteins and exhibits hepatotoxic properties in *Acanthaster planci*, the species from which this toxin was first described [56–57]. Additionally, plancitoxin-1 toxin was also previously identified in several nemertean species and the jellyfish *S*. *meleagris* [9,58]. A single transcript with a full-length ORF was identified that encoded a peptide exhibiting high identity with venom toxin-like peptide-6 (GenBank accession number ABR21046.1) and venom protein 2 (ABR21036.1), which were both isolated from the venom of the scorpion *Mesobuthus eupeus* (Fig 7). The sequence of the transcript comprises 71 amino acids and displays 45% and 44% identity, respectively, with these two scorpion toxins. However, the functions of these venom toxins in envenomation remain unknown. Two transcripts similar to Ci-120 and Ci-80a were also characterized (S4 Fig). Ci-120 and Ci-80a are both venom proteins identified from the parasitic wasp *Chelonus inanitus* [40]. The Ci-120 protein, which may affect proteoglycan metabolism, exhibits high sequence similarity to alpha-N-acetylglucosaminidase. Ci-80a is a member of the papain family, and its role in envenomation remains to be determined. In addition, we identified a transcript encoding a protein similar to venom dipeptidyl peptidase 4(DPP4) isoform X2 (S5 Fig). DPP4 has been widely identified in snake venoms [59], suggesting an important role in envenomation. Venom DPP4 is involved in the processing of venom peptides and may also play a role in cardiovascular disorders caused by the venom [60–61].

**Fig 8:**
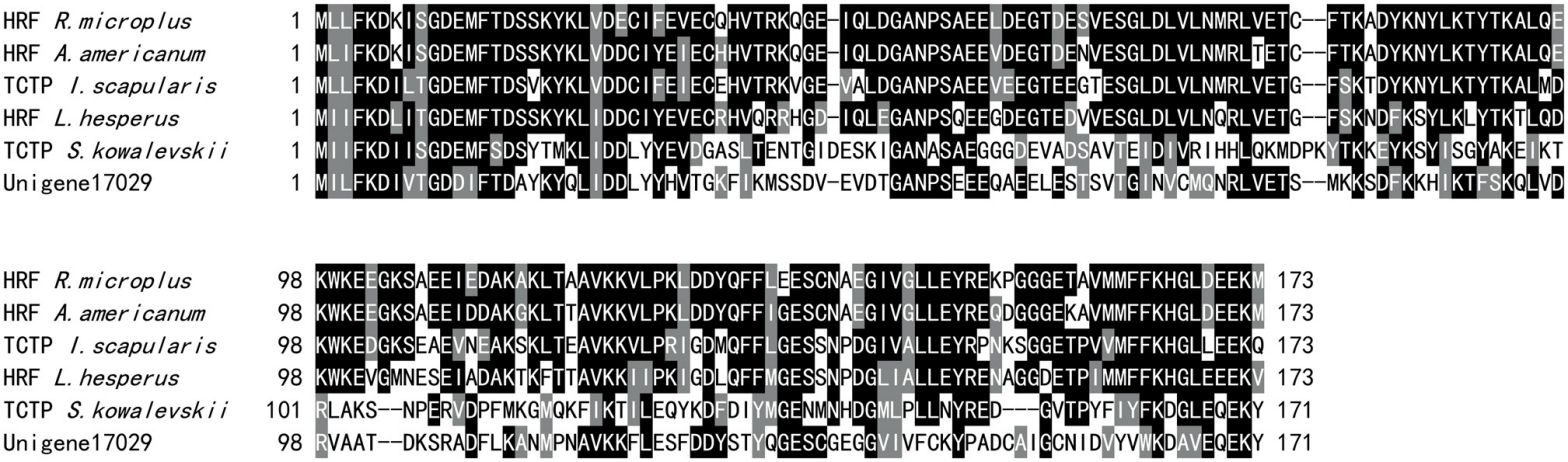
Alignment of translationally controlled tumor protein (or histamine-releasing factor). The aligned sequences are as follows: *Rhipicephalus microplus* histamine release factor (AAY67698), *Amblyomma americanum* histamine release factor (AAY67700), *Latrodectus hesperus* histamine release factor (ADV40083), *Saccoglossus kowalevskii* TCTP (XP_002740226) and *Ixodes scapularis* TCTP (AAY66972). Black and gray indicate amino acids that are identical or highly conserved across all aligned sequences, respectively.

In addition to these toxin-like candidates, some other putative venom components were also identified in the transcriptome of *C*. *capillata* tentacle by BLAST searches. Translationally controlled tumor protein (TCTP) has been identified in the gland secretions of ticks and mites [62–63]. TCTP is also called histamine-releasing factor (HRF) and has been reported as a venom toxin in several spider species [36]. In our transcriptome of *C*. *capillata*, a transcript exhibiting significant similarity with TCTP (or HRF) was identified (Fig 8). TCTP induces histamine release in basophil leukocytes and is one of the molecules responsible for histamine-associated symptoms. Interestingly, local tissue edema, the main effect of histamine release, always occurs after jellyfish stinging. Therefore, we suggest that TCTP in *C*. *capillata* tentacle may play a role in histamine release during jellyfish envenomation.

We also identified a transcript exhibiting significant similarity to various angiotensin-converting enzyme-like (ACE-like) proteins and a transcript similar to endothelin-converting enzyme 1-like (ECE 1-like) proteins (S6 Fig). ACE and ECE are both metalloproteases. ACE plays a role in converting angiotensin I to angiotensin II, which can induce vasoconstriction and elevate blood pressure. ECE is involved in the processing of ET-1, a potent vasoconstrictor, from its inactive precursor. Expression of ACE (or ACE-like) and ECE proteins in the venoms of several species of cone snails and wasps has been reported [40,64]. However, this is the first report of the presence of these molecules in jellyfish. Cardiovascular toxicity is the major bioactivity of jellyfish venoms. We previously demonstrate that *C*. *capillata* venom induces marked vasoconstriction [65]. Therefore, the identification of ACE and ECE-1 in *C*. *capillata* tentacle strongly suggests that these molecules might contribute to the disruption of cardiovascular function caused by jellyfish venoms.

**Fig 9:**
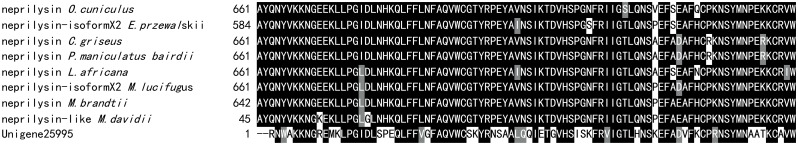
Multiple sequence alignment of the amino acid sequence of unigene 25995 with other known neprilysin or neprilysin-like proteins. The aligned sequences are as follows: *Oryctolagus cuniculus* neprilysin (NP_001095155), *Equus przewalskii* neprilysin isoform X2 (XP_008519227), *Loxodonta africana* neprilysin (XP_003416187), *Cricetulus griseus* neprilysin (XP_007625698), *Peromyscus maniculatus bairdii* neprilysin (XP_006972674), *Myotis lucifugus* neprilysin isoform X2 (XP_006090392), *Myotis brandtii* neprilysin (EPQ12789) and *Myotis davidii* neprilysin-like (XP_006769958).

Additionally, a transcript that exhibited significant sequence similarity to neprilysin (or neprilysin-like protein) was identified (Fig 9). Neprilysin is a zinc-dependent metalloprotease with affinity for a broad range of physiological targets, including natriuretic, vasodilatory and neuro peptides [66]. Notably, neprilysin has been identified in the venoms of several species of spiders and snakes and is likely correlated with neurotoxicity, potentially due to its activity in the inactivation of peptide transmitters and their modulators [66]. Interestingly, jellyfish venom comprises neurotoxins that immediately paralyze prey. We have also observed this neurotoxic activity in *C*. *capillata* venom. Therefore, neprilysin (or neprilysin-like protein) may also play a role in *C*. *capillata* envenomation, but its precise role requires further study.

We also identified four transcripts exhibiting similarity to the ectonucleotide pyrophosphatase/phosphodiesterase family (Table 3). Phosphodiesterase activity has been described in many snake venoms [67–68]. A phosphodiesterase family member has been identified as a toxin in the jellyfish *S*. *meleagris* [9]. However, the contributions of these proteins to the poisoning mechanism are poorly understood. Phosphodiesterases might exhibit inhibitory activity on ADP-induced platelet aggregation and contribute to hemostatic disturbances. Therefore, the discovery of an ectonucleotide pyrophosphatase/phosphodiesterase family in *C*. *capillata* tentacle implies that this superfamily might be constituents of jellyfish venom and their roles in envenomation deserve further investigation.

In addition, sequences with similarity to vascular endothelial growth factors (VEGFs) were also identified (Table 3). VEGFs, which contain several subclasses, have been described as minor venom constituents in the venom glands of several snake species [28–29]. Among these transcripts, two sequences exhibited similarity to VEGF-A, which has been reported to induce vasodilation and potently increase vascular permeability. It can also promote tachycardia and hypotension and diminish cardiac output [69]. We observed these symptoms when *C*. *capillata* venom was injected in rats. Therefore, this family might also play a role in *C*. *capillata* envenomation.

In addition to the molecules mentioned above, we also identified several other possible venom components, including lysosomal acid lipases (LALs), alkaline phosphatase, dipeptidyl peptidase 3 and ectonucleoside triphosphate diphosphohydrolase (Table 3). These transcripts have also been previously reported as atypical venom components in some venomous animals [26,67], and none of these transcripts have been previously described in jellyfish. However, their contributions to the bioactivities of jellyfish venom require experimental verification.

### Transcripts relevant to degenerative diseases

In addition to putative toxin transcripts, disease-related transcripts were also identified (data not shown). Among them, the most surprising were transcripts relevant to three nervous system diseases, Huntington’s disease (HD), Alzheimer's disease (AD) and Parkinson's disease (PD).

**Fig 10:**
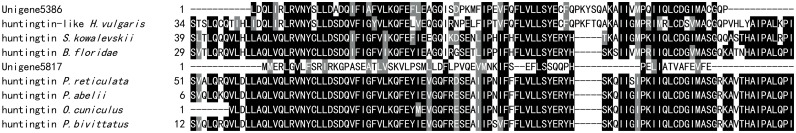
Multiple sequence alignment of the amino acid sequence of unigenes 5396 and 5817 with those of other known huntingtin proteins. The aligned sequences are as follows: *Hydra vulgaris* huntingtin-like (XP_004206850), *Saccoglossus kowalevskii* huntingtin (XP_006822985), *Poecilia reticulate* huntingtin isoform X 5 (XP_008406720), *Pongo abelii* huntingtin (XP_002814571), *Oryctolagus cuniculus* huntingtin (XP_008248081), *Python bivittatus* huntingtin (XP_007432531) and *Branchiostoma floridae* huntingtin (ABP04240).

We characterized 476 unigenes involved in the pathway of Huntington’s disease, the fourth largest group in KEGG annotation. HD is an inherited neurodegenerative disease [70]. In this study, transcripts homologous to genes closely related to HD, including Huntingtin, Huntingtin-interacting protein 1 and Huntingtin-interacting protein K, were all identified (S3 Table). Sequence analysis revealed that the Huntingtin genes were highly conserved in various species (Fig 10). We also identified two transcripts encoding proteins similar to presenilin-2 (S7 Fig). Mutations in the genes encoding presenilin-1 and presenilin-2 are responsible for early-onset autosomal dominant Alzheimer's disease, the most frequent degenerative dementia among the elderly [71]. There are no effective treatments for HD or AD. Transcripts encoding proteins homologous to Parkinson disease-related proteins, including Parkinson protein 7 (protein DJ-1) and Parkinson disease 7 domain-containing protein 1, were also identified in the transcriptome of *C*. *capillata* tentacle (S3 Table).

This is the first description of the expression of these nervous system disease-associated genes in jellyfish species. Interestingly, in a previous study of the genome of *Hydra magnipapillata*, genes associated with nervous system diseases, including HD and AD, were also identified [72]. Therefore, these results strongly suggest that degenerative disease-related genes are highly conserved from invertebrates to vertebrates. These findings also suggest the great potentials of these marine invertebrates as models in the study of degenerative diseases.

## Conclusions

This study contributes to a more comprehensive view of the origin and functional diversity of venom proteins in jellyfish, provides a foundation and valuable resource for further investigations of bioactive components, and promotes the general development of jellyfish resources.

## Supporting Information

S1 Fig The size distributions of assembled contigs and unigenes. (A) Length distribution of contigs. (B) Length distribution of unigenes. The number under the x-axis indicates the length range (e.g., ‘300’ indicates a length range of (200, 300), whereas‘>3000’ indicates a length range longer than 3,000 bp.). The y-axis is in logarithmic scale. The number above each bar indicates the total number of sequences falling in this length range.
(TIF)

S2 Fig GO categories of the unigenes. The GO categories shown in the x-axis were grouped into three main categories: biological process, cellular component and molecular function. The right y-axis indicates the number of annotated unigenes in each sub-category, and the left y-axis indicates the percentage of total unigenes in that sub-category.
(TIF)

S3 Fig COG classification of the unigenes. 6,202 unigenes (12.3% of the total) were annotated and classified into 25 COG functional categories.
(TIF)

S4 Fig **(A)** Alignment of the amino acid sequence of unigene 4165 with parasitic wasp *Chelonus inanitus* venom protein Ci-120 (CBM69278). **(B)** Alignment of the amino acid sequence of unigene 49242 with parasitic wasp *Chelonus inanitus* venom protein Ci-80a (CBM69275).
(TIF)

S5 Fig Alignment of venom dipeptidyl peptidase 4. The aligned sequences are as follows: *Nasonia vitripennis* venom dipeptidyl peptidase 4 isoform X2 (XP_008202161), *Ceratosolen solmsi marchali* venom dipeptidyl peptidase 4 isoform X2 (XP_011494919) and *Cerapachys biroi* venom dipeptidyl peptidase 4 isoform X2 (XP_011336230). Black and gray indicate amino acids that are identical or highly conserved across all aligned sequences, respectively.
(TIF)

S6 Fig **(A) Multiple sequence alignment of the amino acid sequence of unigene 7297 with other known ACE and ACE-like proteins.** The aligned sequences are as follows: *Strongylocentrotus purpuratus* ACE-like isoform 1(XP_003724612), *Hydra vulgaris* ACE-like (XP_004208490), *Colius striatus* ACE (XP_010200493), *Acanthisitta chloris* ACE (XP_009082259), *Notothenia coriiceps* ACE (XP_010793898), *Pseudopodoces humilis* ACE (XP_005532492) and *Homo sapiens* ACE 1(EAW94314). **(B) Multiple sequence alignment of the amino acid sequence of unigene 29942 with other known ECE and ECE-like proteins.** The aligned sequences are as follows: *Hydra vulgaris* ECE 1-like (XP_004211607), *Apis florea* ECE 2-like (XP_003693348), *Hydra vulgaris* ECE (AAD46624) and *Metaseiulus occidentalis* ECE 2-like (XP_003743930). Black and gray indicate amino acids that are identical or highly conserved across all aligned sequences, respectively.
(TIF)

S7 Fig Multiple sequence alignment of the amino acid sequence of the unigenes 7582 and 8824 with other known presenilin 2 sequences. The aligned sequences are as follows: *Xenopus tropicalis* presenilin 2 (NP_001017181), *Lepisosteus oculatus* presenilin-2-like (XP_006638781), *Octodon degus* presenilin-2-like isoform X2 (XP_004626898), *Takifugu rubripes* presenilin-2-like (XP_003972298), *Stegastes partitus* presenilin-2 (XP_008297105), *Taeniopygia guttata* presenilin-2 (XP_002197681) and *Callorhinchus milii* presenilin-2 (XP_007891888).
(TIF)

S1 Table Functional annotations of 50,536 unigenes by Blast alignments to several public databases. (XLSX)

S2 Table Categorization of unigenes into KEGG biochemical pathways. (DOCX)

S3 Table Transcripts relevant to degenerative diseases. (DOCX)
