# Supplementary figures and images for: Global Transcriptome Analysis of the Tentacle of the Jellyfish Cyanea capillata Using Deep Sequencing and Expressed Sequence Tags: Insight into the Toxin- and Degenerative Disease-Related Transcripts
Source: PLoS One. 2015 Nov 9;10(11):e0142680. doi: 10.1371/journal.pone.0142680 (PMC4638339; doi:10.1371/journal.pone.0142680)

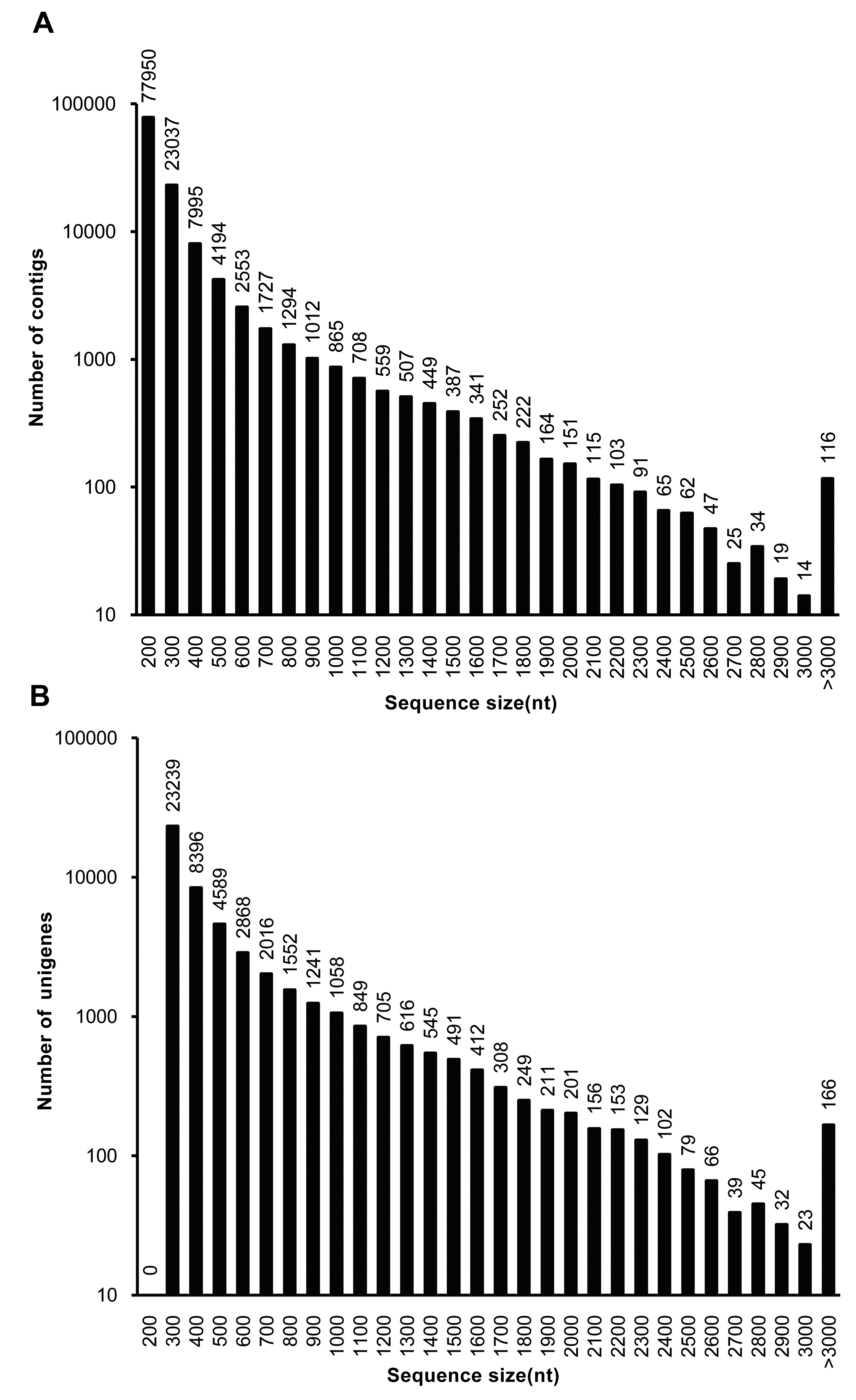

Supplement: S1 Fig — (A) Length distribution of contigs. (B) Length distribution of unigenes. The number under the x-axis indicates the length range (e.g., ‘300’ indicates a length range of (200, 300), whereas‘>3000’ indicates a length range longer than 3,000 bp.). The y-axis is in logarithmic scale. The number above each bar indicates the total number of sequences falling in this length range. (TIF) [file pone.0142680.s001.tif]

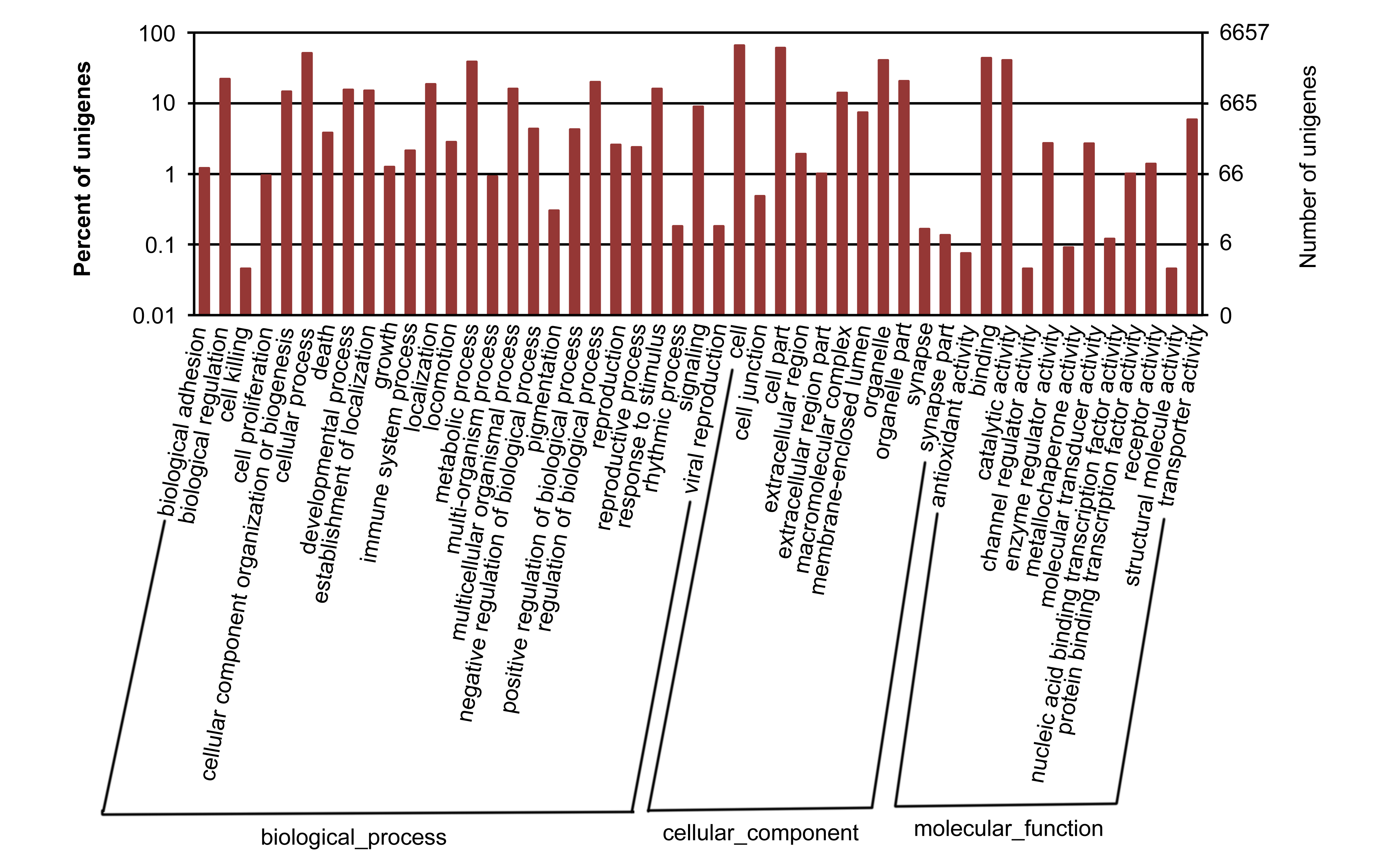

Supplement: S2 Fig — The GO categories shown in the x-axis were grouped into three main categories: biological process, cellular component and molecular function. The right y-axis indicates the number of annotated unigenes in each sub-category, and the left y-axis indicates the percentage of total unigenes in that sub-category. (TIF) [file pone.0142680.s002.tif]

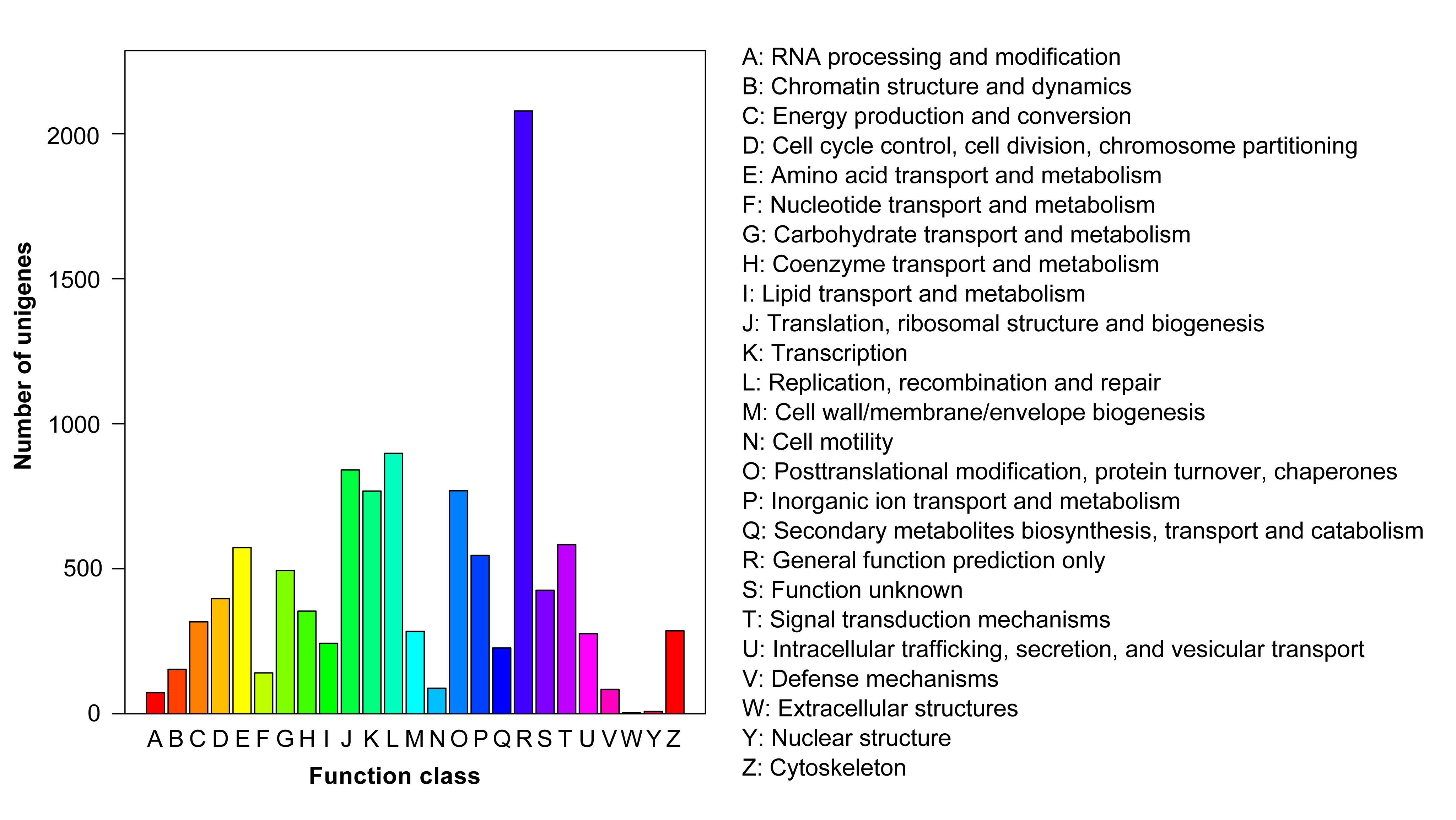

Supplement: S3 Fig — 6,202 unigenes (12.3% of the total) were annotated and classified into 25 COG functional categories. (TIF) [file pone.0142680.s003.tif]

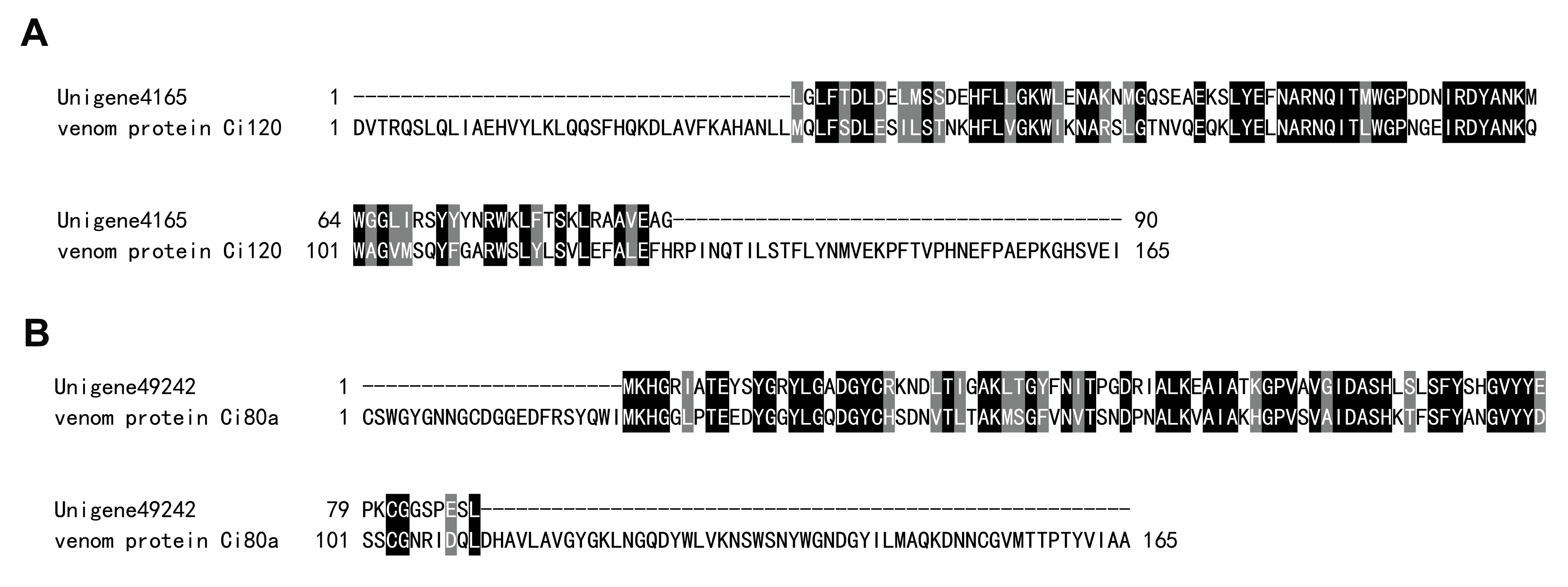

Supplement: S4 Fig — (A) Alignment of the amino acid sequence of unigene 4165 with parasitic wasp Chelonus inanitus venom protein Ci-120 (CBM69278). (B) Alignment of the amino acid sequence of unigene 49242 with parasitic wasp Chelonus inanitus venom protein Ci-80a (CBM69275). (TIF) [file pone.0142680.s004.tif]

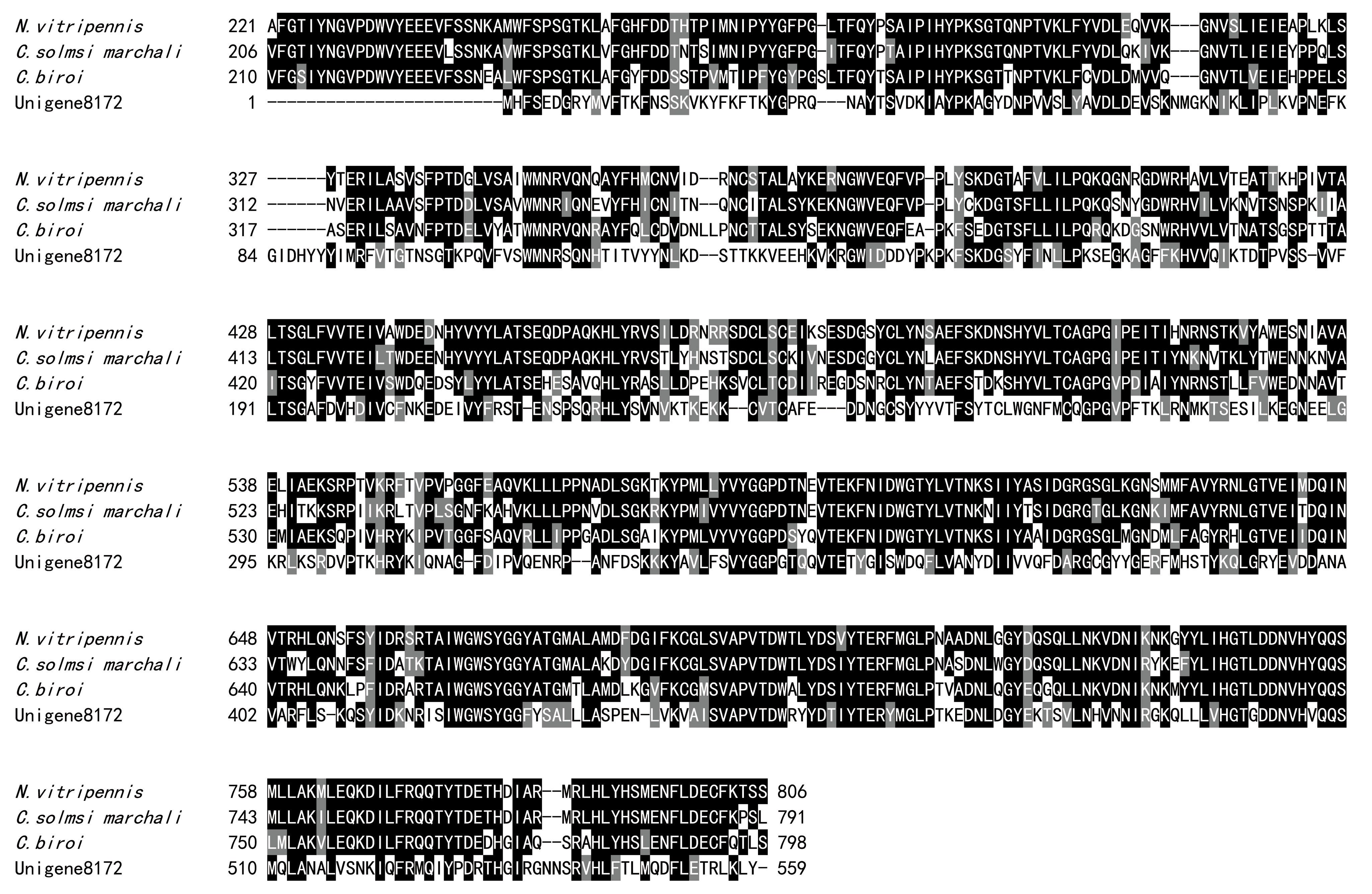

Supplement: S5 Fig — The aligned sequences are as follows: Nasonia vitripennis venom dipeptidyl peptidase 4 isoform X2 (XP_008202161), Ceratosolen solmsi marchali venom dipeptidyl peptidase 4 isoform X2 (XP_011494919) and Cerapachys biroi venom dipeptidyl peptidase 4 isoform X2 (XP_011336230). Black and gray indicate amino acids that are identical or highly conserved across all aligned sequences, respectively. (TIF) [file pone.0142680.s005.tif]

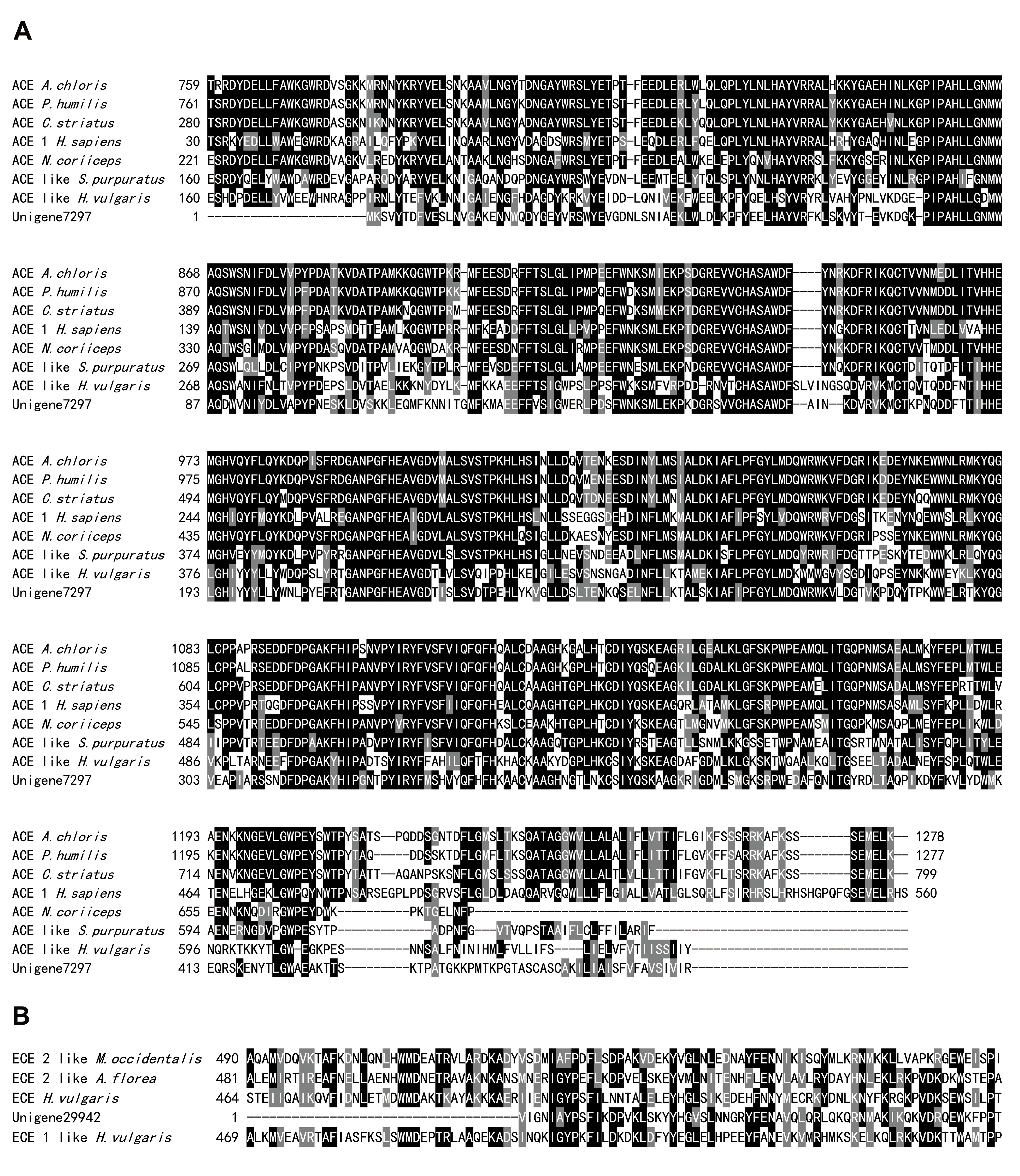

Supplement: S6 Fig — (A) Multiple sequence alignment of the amino acid sequence of unigene 7297 with other known ACE and ACE-like proteins. The aligned sequences are as follows: Strongylocentrotus purpuratus ACE-like isoform 1(XP_003724612), Hydra vulgaris ACE-like (XP_004208490), Colius striatus ACE (XP_010200493), Acanthisitta chloris ACE (XP_009082259), Notothenia coriiceps ACE (XP_010793898), Pseudopodoces humilis ACE (XP_005532492) and Homo sapiens ACE 1(EAW94314). (B) Multiple sequence alignment of the amino acid sequence of unigene 29942 with other known ECE and ECE-like proteins. The aligned sequences are as follows: Hydra vulgaris ECE 1-like (XP_004211607), Apis florea ECE 2-like (XP_003693348), Hydra vulgaris ECE (AAD46624) and Metaseiulus occidentalis ECE 2-like (XP_003743930). Black and gray indicate amino acids that are identical or highly conserved across all aligned sequences, respectively. (TIF) [file pone.0142680.s006.tif]

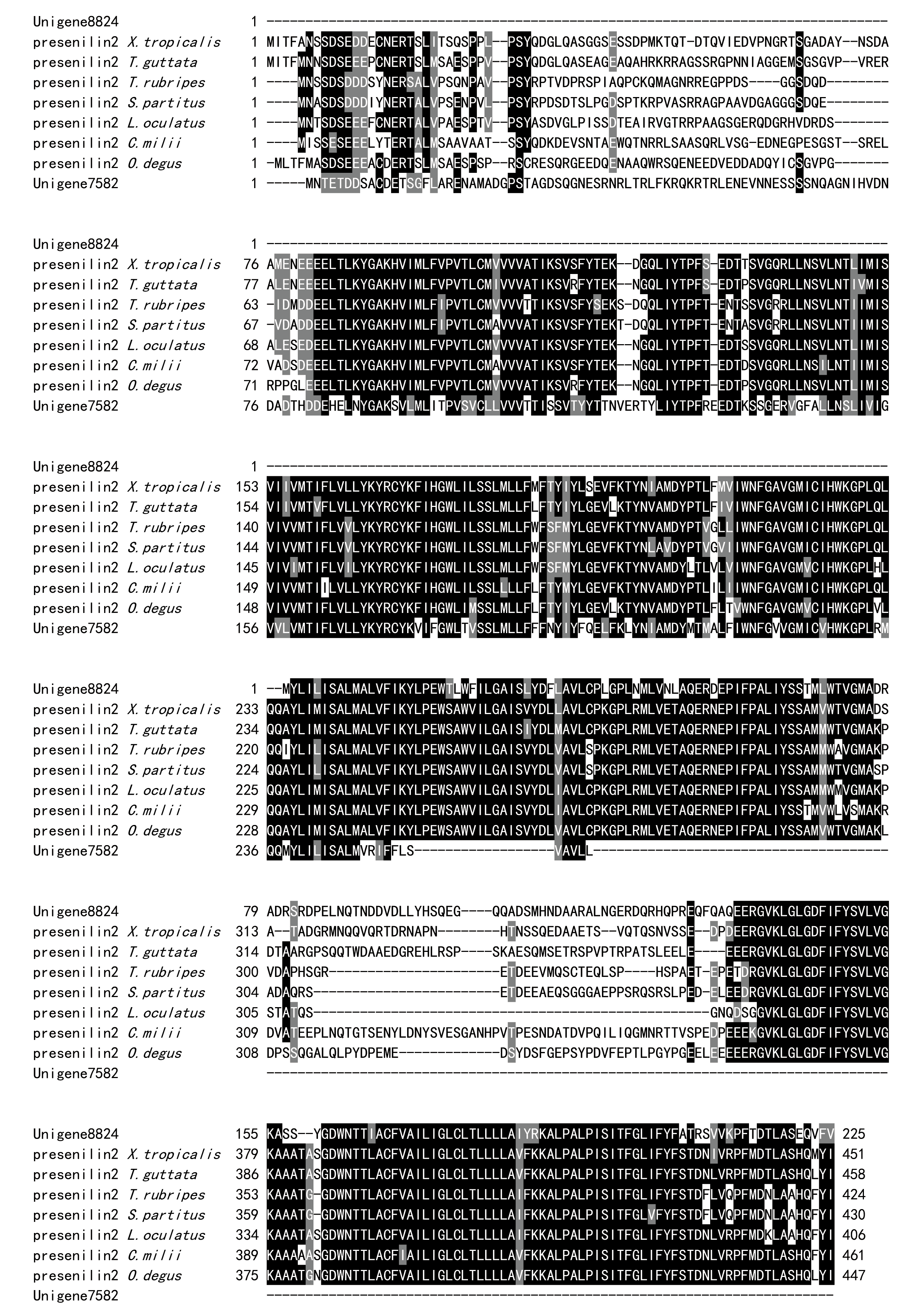

Supplement: S7 Fig — The aligned sequences are as follows: Xenopus tropicalis presenilin 2 (NP_001017181), Lepisosteus oculatus presenilin-2-like (XP_006638781), Octodon degus presenilin-2-like isoform X2 (XP_004626898), Takifugu rubripes presenilin-2-like (XP_003972298), Stegastes partitus presenilin-2 (XP_008297105), Taeniopygia guttata presenilin-2 (XP_002197681) and Callorhinchus milii presenilin-2 (XP_007891888). (TIF) [file pone.0142680.s007.tif]
