# Supplementary material for: Global Transcriptome Analysis of the Tentacle of the Jellyfish Cyanea capillata Using Deep Sequencing and Expressed Sequence Tags: Insight into the Toxin- and Degenerative Disease-Related Transcripts
Source: PLoS One. 2015 Nov 9;10(11):e0142680. doi: 10.1371/journal.pone.0142680 (PMC4638339; doi:10.1371/journal.pone.0142680)
Supplement: S2 Table — (DOCX) [file pone.0142680.s009.docx]

**Table S2. Categorization of unigenes to KEGG biochemical pathways**

| **KEGG categories** | **Number of assigned pathways** | **Number of annotated unigenes** |
| --- | --- | --- |
| **Metabolism** |  |  |
| Carbohydrate Metabolism | 14 | 1029 |
| Energy Metabolism | 3 | 172 |
| Lipid Metabolism | 15 | 716 |
| Nucleotide Metabolism | 2 | 554 |
| Amino Acid Metabolism | 13 | 975 |
| Metabolism of Other Amino Acids | 7 | 249 |
| Glycan Biosynthesis and Metabolism | 12 | 461 |
| Metabolism of Cofactors and Vitamins | 12 | 303 |
| Metabolism of Terpenoids and Polyketides | 3 | 31 |
| Biosynthesis of Other Secondary Metabolites | 2 | 17 |
| Xenobiotics Biodegradation and Metabolism | 3 | 151 |
| **Genetic Information Processing** |  |  |
| Transcription | 3 | 553 |
| Translation | 5 | 921 |
| Folding, Sorting and Degradation | 7 | 963 |
| Replication and Repair | 6 | 381 |
| **Environmental Information Processing** |  |  |
| Membrane Transport | 1 | 118 |
| Signal Transduction | 12 | 1867 |
| Signaling Molecules and Interaction | 4 | 782 |
| **Cellular Processes** |  |  |
| Transport and Catabolism | 5 | 1204 |
| Cell Motility | 1 | 420 |
| Cell Growth and Death | 4 | 676 |
| Cell Communication | 4 | 1046 |
| **Organismal Systems** |  |  |
| Immune System | 15 | 1452 |
| Endocrine System | 7 | 969 |
| Circulatory System | 2 | 471 |
| Digestive System | 9 | 1297 |
| Excretory System | 5 | 419 |
| Nervous System | 5 | 793 |
| Sensory System | 4 | 337 |
| Development | 3 | 382 |
| Environmental Adaptation | 2 | 42 |
| **Human Diseases** |  |  |
| Cancers | 15 | 1843 |
| Immune Diseases | 7 | 148 |
| Neurodegenerative Diseases | 5 | 1143 |
| Cardiovascular Diseases | 4 | 736 |
| Endocrine and Metabolic Diseases | 3 | 76 |
| Infectious Diseases | 17 | 2284 |
