# Supplementary material for: Global Transcriptome Analysis of the Tentacle of the Jellyfish Cyanea capillata Using Deep Sequencing and Expressed Sequence Tags: Insight into the Toxin- and Degenerative Disease-Related Transcripts
Source: PLoS One. 2015 Nov 9;10(11):e0142680. doi: 10.1371/journal.pone.0142680 (PMC4638339; doi:10.1371/journal.pone.0142680)
Supplement: S3 Table — (DOCX) [file pone.0142680.s010.docx]

**Table S3. Transcripts closely relevant to degenerative diseases**

| **Sequence ID** | **Length(bp)/ ORF*** | **Identification (best matched species)** | **E-value** |
| --- | --- | --- | --- |
| **Huntington’s associated** |  |  |  |
| Unigene5386 | 271/N | Huntingtin-like (*Hydra vulgaris*) | 8E-41 |
| Unigene5817 | 251/N | Huntingtin-like (*Hydra vulgaris*) | 1E-23 |
| Unigene10685 | 416/N | Huntingtin-interacting protein 1-like (*Hydra vulgaris*) | 3E-06 |
| Unigene17760 | 813/N | Huntingtin interacting protein K-like (*Hydra vulgaris*) | 5E-22 |
| Unigene30330 | 409/N | Huntingtin-interacting protein 1-related protein-like (*Hydra vulgaris*) | 1E-34 |
| Unigene31086 | 299/N | Huntingtin interacting protein 1-like (*Hydra vulgaris*) | 3E-07 |
| **Alzheimer's associated** |  |  |  |
| Unigene7582 | 981/F | Presenilin-2 (*Xenopus tropicallis*) | 8E-56 |
| Unigene8824 | 1487/F | Presenilin-2 (*Xenopus laevis*) | 7E-77 |
| Unigene22364 | 493/N | Presenilins-associated rhomboid-like protein, mitochondrial (*Tursiops truncatus*) | 6E-30 |
| Unigene41332 | 607/N | Presenilins-associated rhomboid-like protein, mitochondrial-like (*Hydra vulgaris*) | 2E-32 |
| **Parkinson associated** |  |  |  |
| Unigene17149 | 881/F | Parkinson disease 7 domain-containing protein 1-like (*Hydra vulgaris*) | 2E-97 |
| Unigene17703 | 799/F | Parkinson protein 7 (*Xenopus laevis*) | 7E-69 |

*Full-length and not full-length open reading frames (ORFs) are indicated with a “F” and “N” respectively.
